# Cavity Born–Oppenheimer Hartree–Fock Ansatz: Light–Matter Properties of Strongly Coupled Molecular Ensembles

**DOI:** 10.1021/acs.jpclett.3c01842

**Published:** 2023-08-31

**Authors:** Thomas Schnappinger, Dominik Sidler, Michael Ruggenthaler, Angel Rubio, Markus Kowalewski

**Affiliations:** † Department of Physics, Stockholm University, AlbaNova University Center, SE-106 91 Stockholm, Sweden; ‡ Max Planck Institute for the Structure and Dynamics of Matter and Center for Free-Electron Laser Science, Luruper Chaussee 149, 22761 Hamburg, Germany; ¶ The Hamburg Center for Ultrafast Imaging, Luruper Chaussee 149, 22761 Hamburg, Germany; § Center for Computational Quantum Physics, Flatiron Institute, 162 Fifth Avenue, New York, New York 10010, United States; ∥ Nano-Bio Spectroscopy Group, University of the Basque Country (UPV/EHU), 20018 San Sebastián, Spain

## Abstract

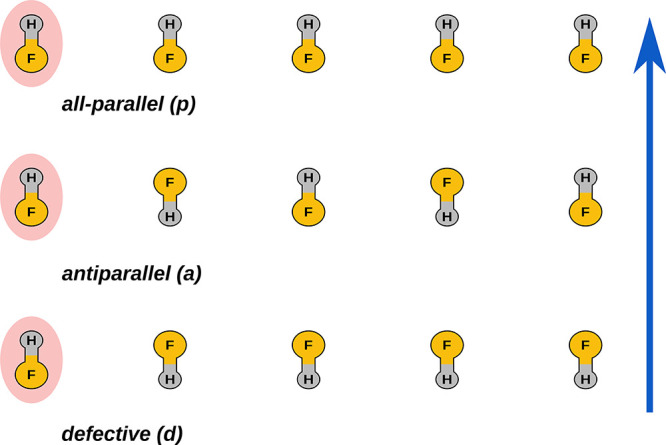

Experimental studies indicate that optical cavities can affect chemical reactions through either vibrational or electronic strong coupling and the quantized cavity modes. However, the current understanding of the interplay between molecules and confined light modes is incomplete. Accurate theoretical models that take into account intermolecular interactions to describe ensembles are therefore essential to understand the mechanisms governing polaritonic chemistry. We present an *ab initio* Hartree–Fock ansatz in the framework of the cavity Born–Oppenheimer approximation and study molecules strongly interacting with an optical cavity. This ansatz provides a nonperturbative, self-consistent description of strongly coupled molecular ensembles, taking into account the cavity-mediated dipole self-energy contributions. To demonstrate the capability of the cavity Born–Oppenheimer Hartree–Fock ansatz, we study the collective effects in ensembles of strongly coupled diatomic hydrogen fluoride molecules. Our results highlight the importance of the cavity-mediated intermolecular dipole–dipole interactions, which lead to energetic changes of individual molecules in the coupled ensemble.

The strong coupling of light and matter within optical cavities provides an innovative way to alter and design matter properties, making it a rapidly evolving field^1−10^ at the intersection of chemistry, quantum optics, and materials science. In polaritonic chemistry, depending on whether the quantized cavity modes are coupled via their characteristic frequencies to electronic or vibrational degrees of freedom of molecules, the situation is described as electronic-strong coupling (ESC) or vibrational-strong coupling (VSC), respectively. Under ESC, it becomes possible to modify the photochemistry/photophysics of molecules including charge transfer processes and electronic spectroscopy, and photoinduced reactions can be influenced.^11−28^ Similarly, for VSC, the vibrational spectra of molecules are altered by the formation of light–matter hybrid states, and even the chemical reactivity of the ground state can be modified.^27,29−34,34−36^ The observed effects of molecular ESC and VSC are often discussed phenomenologically, and understanding of the underlying microscopic and macroscopic physical mechanisms, especially with respect to the effects of VSC, is still incomplete.^6,37−41^ In our recent work^42^ we have shown numerically that the interaction between an optical cavity and ensembles of molecules not only leads to cavity detuning and a change of the optical length but also allows for a local molecular polarization mechanism under strong collective vibrational coupling in the thermodynamic limit. The interplay of microscopic and macroscopic polarization is due to cavity-mediated dipole–dipole interaction. A deeper understanding of this effect may bridge the gap between existing simplified models for a macroscopic ensemble of molecules and experiments. We have been able to study this cavity-mediated dipole–dipole interaction for very large ensembles by using simple Shin–Metiu molecules.^42^ As a next step to go beyond this simple molecular model, we present here a formulation of the well-known Hartree–Fock ansatz in the context of the cavity Born–Oppenheimer approximation (CBOA)^43−45^ derived from the complete nonrelativistic Pauli–Fierz Hamiltonian.^2,46−48^ We refer to the resulting method as cavity Born–Oppenheimer-Hartree–Fock (CBO-HF) approach, and to the best of our knowledge, this is the first wave function-based method to describe strong coupling of real molecules in a cavity in the CBOA framework. The CBO-HF method allows us to study cavity-mediated dipole–dipole interactions for realistic molecular systems. The first part of this Letter describes the CBO-HF formalism. Next, the CBO-HF approach is used to explore the effects of collective cavity-mediated coupling in small ensembles of diatomic hydrogen fluoride (HF) molecules. By explicitly simulating the molecular systems in a self-consistent calculation, we are able to study the interactions within the ensemble beyond what can be captured by scaled single-molecule model Hamiltonians. In the following we study static ensembles of molecules, but we do not address the effects of vibrational resonances.^48−50^ Consistent with recently reported results,^42,51^ we observe non-negligible cavity-induced energy changes at the local molecular level. By a detailed analysis of these energy changes at the microscopic (single-molecule) and macroscopic (molecular ensemble) levels, we can show how the size of the ensemble, the individual molecular orientation, and the change in nuclear configuration can affect these collective interactions.

The physics of a cavity-embedded molecular system is described using the nonrelativistic Pauli–Fierz Hamiltonian,^2,46−48^ which is represented in the length gauge, assuming the dipole approximation and the CBOA.^43−45^ Within the CBOA, the cavity modes and nuclei are considered to be “slow” degrees of freedom compared to the “fast” electrons, and consequently, only the electrons are treated quantum mechanically. In the following, bold symbols denote vectors, and atomic units (*ℏ* = 4π; ε_0_ = *m*_*e*_ = 1) are used throughout the Letter, unless otherwise indicated. For a single-mode cavity, the CBOA Hamiltonian takes the form
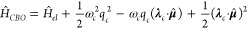
1where
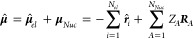
2represents the molecular dipole operator, which is defined by the operators of the *N*_*el*_ electron coordinates ***r*^**, the classic coordinates ***R*** of the *N*_*Nuc*_ nuclei, and the nuclear charges *Z*_*A*_.  is the Hamiltonian for the field-free many-electron system, and the second term defines the harmonic potential introduced by the photon displacement field, with the photon displacement coordinate *q*_*c*_ and ω_*c*_ being the frequency of the cavity mode. The third term of eq 1 describes the dipole coupling between the molecular system and the photon displacement field, which is characterized by coupling strength **λ**_*c*_. The last term is the dipole self-energy (DSE) operator,^6,52^ which is an energy contribution that describes the self-polarization of the molecule–cavity system. Note that the inclusion of the DSE contribution is strictly necessary to obtain a finite polarization and a bounded solution.^53^ In practice, localized and finite basis sets, such as Gaussian basis sets, are used in most quantum chemistry methods. This limits the polarization, and the lack of a stable ground state is not always observed in the numerical calculations. In the following, we will show that the DSE term is not only formally needed but yields intermolecular interactions in an ensemble of molecules. The coupling parameter **λ**_*c*_ for a cavity with an effective mode volume *V*_*c*_ is defined as follows:
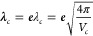
3The unit vector ***e*** denotes the polarization axis of the cavity mode. In the context of a Fabry–Pérot-type cavity, **λ**_*c*_ can be directly related to the electric vacuum field strength ϵ_*c*_ via . Without a loss of generality, we will use this relation to quantify λ_*c*_ by ϵ_*c*_.

As in the standard Hartree–Fock approach, the CBO-HF electronic wave function of an *N*_*el*_-electron molecular system is a Slater determinant of mutually orthonormal spin orbitals φ_*i*_:^54^
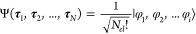
4Here, **τ**_*N*_ is used to denote the complete set of coordinates associated with the *N*th electron, comprised of the spatial coordinate ***r***_*N*_ and a spin coordinate. Note that the *N*_*el*_-electron system described by Ψ can be a single molecule or an ensemble of many molecules. Thus, it is possible to treat cavity-induced interactions and standard Coulomb interactions between molecules in the ensemble in the same way. For the special case of the dilute gas limit, that is, the situation in which the electronic structures of different molecules do not overlap and interact, the ensemble Slater determinant may be replaced by a product^42^ of individual molecular Slater determinants. Note that the displacement coordinate *q*_*c*_ of the electric field mode is treated as a parameter in the CBO-HF ansatz, analogous to the nuclear coordinates, and thus is not part of the wave function.

Using  and Ψ, the CBO-HF energy expectation value *E*_*CBO*_ can be determined using the standard self-consistent field (SCF) procedure:^54^
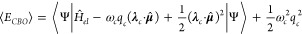
5The resulting energy expectation value *E*_*CBO*_ consists of four energy contributions:
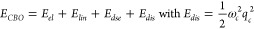
6The detailed derivation of all new energy contributions in the CBO-HF ansatz is given in section S1 of the Supporting Information. The following discussion of these energies is based on Hartree–Fock matrix elements formulated in the basis of orthonormal spin orbitals. The first term *E*_*el*_ contains all Hartree–Fock energy components of the many-electron system^54^ and is only indirectly affected by the cavity via the SCF procedure. The second term *E*_*lin*_ describes the linear part of the light–matter coupling and is obtained from the dipole coupling among the photon displacement field, the electrons, and the nuclei. It can be written as a sum of one-electron integrals formulated in terms of spin orbitals φ_*i*_ and a parametric nuclear contribution:
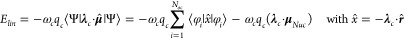
7The remaining component *E*_*dse*_ can be decomposed into a purely electronic term, a mixed electron–nuclear term, and a pure nuclear contribution:
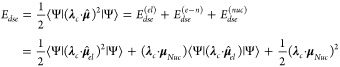
8To simplify the discussion of the cavity-induced modifications, the classical nuclei are arranged so that their contributions to the total dipole moment are zero (**μ**_*Nuc*_ = 0, center of charge). Thus, the nuclear contribution in eq 7 as well as *E*_*dse*_^(*e*–*n*)^ and *E*_*dse*_^(*nuc*)^ are zero by definition since all nuclear contributions are included in our CBO-HF implementation. More details on the latter two can be found in section S1 of the Supporting Information. The pure electronic contribution *E*_*dse*_^(*el*)^ contains a squared electronic position operator , which is a two-electron operator but can be decomposed into one-electron contributions and a two-electron contributions (for details see eq S7 in the Supporting Information):^55−58^
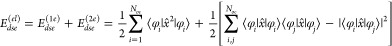
9The one-electron *E*_*dse*_^(1*e*)^ term retains the quadratic nature of the *x̂*^2^ operator and behaves like scaled quadrupole tensor elements and describes a localized energy contribution. The expansion of the two-electron part, *E*_*dse*_^(2*e*)^ in terms of spin orbitals, follows a logic similar to that of the derivation of the Coulomb and exchange integrals in the regular Hartree–Fock ansatz. The terms here can be factorized and further decomposed into a dipole–dipole interaction component, *E*_*dse*_^(2*J*)^, and an exchange-like component, *E*_*dse*_^(2*K*)^. This exchange-like quantity *E*_*dse*_^(2*K*)^ vanishes if φ_*i*_ and φ_*j*_ have no spatial overlap and therefore describes a localized interaction:
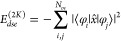
10The *E*_*dse*_^(2*J*)^ part can be rewritten as a product of the scaled electronic dipole moments:
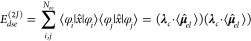
11Unlike *E*_*dse*_^(2*K*)^, the dipole–dipole interaction *E*_*dse*_^(2*J*)^ does not require a spatial overlap of spin orbitals φ_*i*_ and φ_*j*_ and thus results in a delocalized interaction. These two properties are of special interest when ensembles of molecules are described. The last energy contribution *E*_*dis*_ in eq 6 is the energy resulting from the photon displacement field.^52^

By replacing the electronic dipole moment  of the total ensemble with the sum over the individual molecular dipole moments in eq 11 we obtain
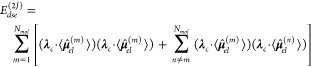
12where the summations run over all molecules *N*_*mol*_ in the ensemble. The first term is the local or intramolecular contribution to *E*_*dse*_^(2*J*)^ for each individual molecule *m*, while the second product describes the interaction of the molecule *m* with all other molecules in the ensemble. This intermolecular interaction depends only on the orientation and size of the individual dipole moments, but not on their distance. This molecular dipole–dipole interaction term *E*_*dse*_^(2*J*)^ in combination with *E*_*dse*_^(*nuc*)^ is commonly used as a first-order approximation^59,60^ of the DSE energy. Note that the full *E*_*dse*_ can also be approximated with the help of permanent dipole moments and transition dipole moments in a resolution of identity approach by summing over excited-electronic states.^22,27,62^

By solving eq 5 with an SCF approach, *E*_*CBO*_ is minimized for a given configuration of classic nuclei and a fixed photon displacement coordinate (parametric photon field). The ground state for the combined electronic–photonic subsystem is obtained by minimizing *E*_*CBO*_ with respect to the photon displacement coordinate, which leads to the following expression:
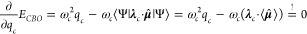
13The resulting minimum of *E*_*CBO*_ is
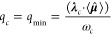
14Because we work in the length gauge, the total electric field (52) is
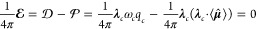
15where  is the displacement field and  is the polarization. Requiring that the transverse electric field vanishes in the ground state,  also leads to eq 14. This demonstrates that minimizing *E*_*CBO*_ with respect to *q*_*c*_ is equivalent to fulfilling the zero transverse electric field condition^6,52^ in the semiclassical limit, which guarantees a nonradiating ground state. From eq 15 we also find that *E*_*dis*_ + *E*_*lin*_ + *E*_*dse*_ = , which is in agreement with Maxwell’s equations.^52^ Thus, by invoking the CBOA, we discard the magnetic contribution to the photonic energy^63^

The main aim of this work is to study an ensemble of well-separated molecules interacting with the cavity field. We thus assume that the wave functions of different molecules in the ensemble do not overlap and that the Coulomb interactions between them is negligible. By satisfying the zero transversal electric field condition (eq 14) for such a molecular ensemble, the photon displacement coordinate *q*_min_ becomes a function of the total ensemble dipole moment. Thus, *E*_*lin*_, *E*_*dis*_, and *E*_*dse*_ are not exclusively dependent on local molecular properties, but rather on the total ensemble.

The CBO-HF method has been implemented in the Psi4NumPy environment,^64^ which is an extension of the PSI4^65^ electronic structure package. All calculations were performed using the aug-cc-pVDZ basis set,^66^ and the geometry of the isolated single HF molecule has been optimized at the Hartree–Fock level of theory. Note that we have not reoptimized the geometries of the molecular systems in the cavity; as such, our calculations do not account for any geometry relaxation effects originating from the presence of the cavity. In all CBO-HF calculations performed in this work, we consider a single mode, lossless cavity. We keep the collective coupling strength **λ**_*c*_ constant by applying a scaling factor of  to obtain a fixed Rabi splitting for different ensemble sizes and treat λ_0_ as a tunable coupling parameter:
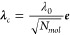
16Here λ_0_ is equivalent to λ_*c*_ in eq 3 in the single-molecule case. As a result, we increase the mode volume *V*_*c*_ of the cavity, but by including more molecules, we keep the average density of molecules *N*_*mol*_/*V*_*c*_ fixed. For *N*_*mol*_ ≫ 1, we would be approaching the thermodynamic limit. Since the main objective of this work is to demonstrate the CBO-HF ansatz and to understand how the different energy contributions to the cavity-mediated interaction under VSC behave when increasing the size of the ensemble but keeping *N*_*mol*_/*V*_*c*_ fixed, it is sufficient to simulate small ensembles. In this work, we restrict ourselves to up to eight HF molecules. We use an artificially increased coupling strength λ_0_ in the range of 0.004–0.04, which corresponds to effective mode volumes (eq 3) in the single-molecule case as large as 125.27 nm^3^ (for λ_0_ = 0.004) or as small as 1.25 nm^3^ (for λ_0_ = 0.04). To refer to a more intuitive physical quantity, the unscaled coupling strength λ_0_ is quantified in this work by the vacuum electric field strength ϵ_*c*_. The fundamental cavity frequency ω_*c*_ is set to be identical with the first vibrational mode of the uncoupled HF molecule, which is 4467 cm^–1^ for the chosen level of theory. This value of ω_*c*_ is chosen arbitrarily in our static setup, and vibrational resonance effects are not present in the model. All calculations were performed in a reproducible environment using the Nix package manager together with NixOS-QChem^67^ (commit f5dad404) and Nixpkgs (nixpkgs, 22.11, commit 594ef126).

In this work, we study fixed ensembles of perfectly aligned, well-separated HF molecules in an optical cavity. We chose HF because of its large permanent dipole moment of 1.8 D, which is advantageous for the interaction between the molecule and the cavity mode, but its polarizability is small with 0.8 Å^3^.^68^ Furthermore, the HF simulation results directly extend our previous results in ref (42), whereas the molecular setup connects to earlier *ab initio* studies on (collective) electronic strong coupling.^51^ To define these ensembles, the optimized structure of a single HF molecule is replicated *N*_*mol*_ times. All of these replicas are separated by 800 Å and placed inside the cavity to avoid interactions via longitudinal electric fields. In general, three different orientations of the molecular HF ensembles are studied in this work and are visualized in Figure 1. In the first orientation, called *all-parallel*, the HF molecules are aligned parallel to the cavity mode polarization axis. In the *antiparallel* case, the *N*_*mol*_ HF molecules are pairwise antiparallel, resulting in the ensemble dipole moment being zero (even number of molecules) or equal to **the dipole moment** of a single HF (odd *N*_*mol*_). The third configuration of the ensemble, labeled *defective*, represents the situation where the dipole moments of *N*_*mol*_ – 1 HF molecules point in the opposite direction to the remaining molecule. Note that for all three ensemble configurations, the individual dipole moment vectors are aligned with the cavity polarization axis and the zero transverse electric field condition (eq 14) is satisfied for the entire ensemble. This aligned orientation is not the energetically most favorable configuration for the individual molecule (see the Supporting Information section S2 for a detailed discussion of the single-molecule situation), but it allows us to set an upper bound on all effects, as it guarantees the maximum molecular cavity interaction.

**Figure 1:**
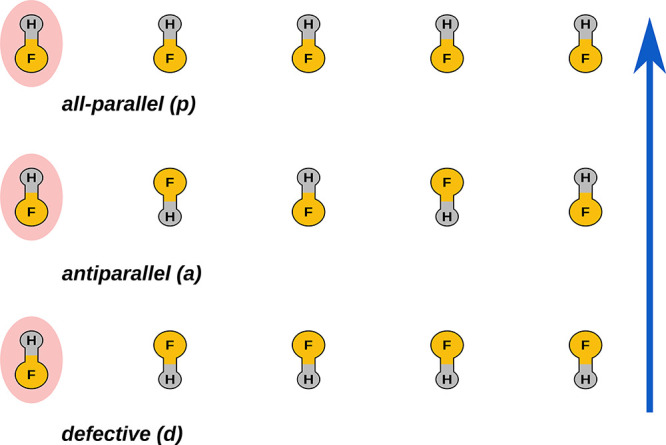
Sketch of the *all-parallel* (p) orientation, the *antiparallel* (a) configuration, and the *defective* (d) orientation shown for an ensemble of five HF molecules as an example. All molecules are separated by a distance of 800 Å. The scan along the bond length is performed for the HF molecule highlighted in red, and the cavity polarization axis is shown in blue. Note that except for the *defective* case, the choice of the highlighted molecule is arbitrary.

All calculations are carried out with rescaled values of **λ**_*c*_ (eq 16). Analogous calculations without rescaling can be found in the Supporting Information (see Figures S5 and S6 in section S3). The energy change of the *all-parallel* ensembles induced by the interaction with the cavity, as well as the underlying energy components, is visualized in Figure 2 as a function of the size of ensemble *N*_*mol*_.

**Figure 2:**
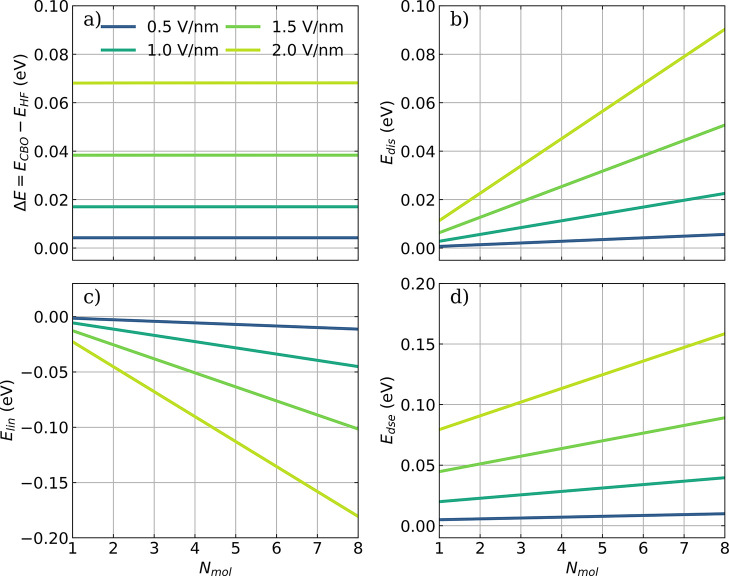
Influence of the cavity interaction on the collective energy of different ensemble sizes and vacuum-field strengths ϵ_*c*_ of HF molecules in the *all-parallel* configuration. (a) The energy difference Δ*E* between *E*_*CBO*_ and the field-free energy *E*_*HF*_, (b) the potential *E*_*dis*_ introduced by the cavity-mediated displacement field, (c) the contribution of the linear dipole coupling *E*_*lin*_, and (d) the DSE contribution *E*_*dse*_ for optimized *q*_*min*_ as a function of *N*_*mol*_. Individual dipole moments are aligned with the cavity polarization axis, and a cavity frequency ω_*c*_ of 4467 cm^–1^ is used. The strength of the cavity field ϵ_*c*_ increases from 0.5 to 2.0 V nm^–1^ (color-coded). The coupling strength **λ**_*c*_ used is rescaled according to eq 16.

Let us first consider how the different total (ensemble) energies behave and what we can learn from them. For simplicity, we focus on the *all-parallel* configuration. In Figure 2a, we see that the proposed rescaling with an increasing ensemble size of the coupling, i.e., the mode volume *V*_*c*_ ∝ *N*_*mol*_, keeps the light and light–matter interaction energy constant. That is, on the total energy level, we see that the thermodynamic limiting procedure is well-behaved, and we expect that approximately also for *N*_*mol*_ ≫ 1 we find such an energy difference. From a perspective of total energy contribution, one might be tempted to conclude that the photon and photon–matter interaction contribution can be safely ignored since *E*_*el*_ increases linearly with *N*_*mol*_ and hence dominates. If we, however, in a next step consider the different contributions of eq 6, we see that even for the total-ensemble energy, a delicate balancing of macroscopically scaling energy contributions appears. Indeed, in Figure 2b, we see that the energy of the displacement field increases linearly even if we rescale the coupling strength. This approximately linearly increasing term is countered by *E*_*lin*_. That *E*_*lin*_ contributes negatively is simple to understand, since the displacement field (interpreted as a constant external field) allows lowering the total energy by separating particles of different charges. Without *E*_*dse*_ depicted in Figure 2d, we would find the well-known result that the linear interaction would dissociate and ionize any bound system regardless of the coupling strength^52,53^ in the large *N*_*mol*_ limit. We can thus conclude that in order to describe an ensemble of molecules from first principles, the dipole self-energy term *E*_*dse*_ is needed to find a stable and physical result.

So far, we have discussed the effect of the collective coupling on the total molecular ensemble. However, the main question in polaritonic chemistry is how a collectively coupled ensemble can influence individual molecules. In the next step, we will thus analyze the energy changes at the level of a single molecule, which arise as a result of the collective interactions of the entire ensemble. Such a local perspective is possible since the ensemble CBO-HF density matrix is a block diagonal, and individual blocks can be used to create partial density matrices for each molecular subsystem. These partial density matrices can be combined with the ensemble Hamiltonian to calculate local energies (per molecule) and combine them pairwise to calculate the interaction between the molecules (see eq 12). This part of the DSE is denoted *inter E*_*dse*_, while all other contributions to the DSE are summed and labeled *local E*_*dse*_. The individual energy obtained per molecule is equivalent to the eigenvalues of the cavity Hartree equation in our previous work.^42^ The change in individual molecular energy induced by the interaction with the cavity as well as the underlying energy components is visualized in Figure 3 as a function of the size of the *all-parallel* ensemble *N*_*mol*_.

**Figure 3:**
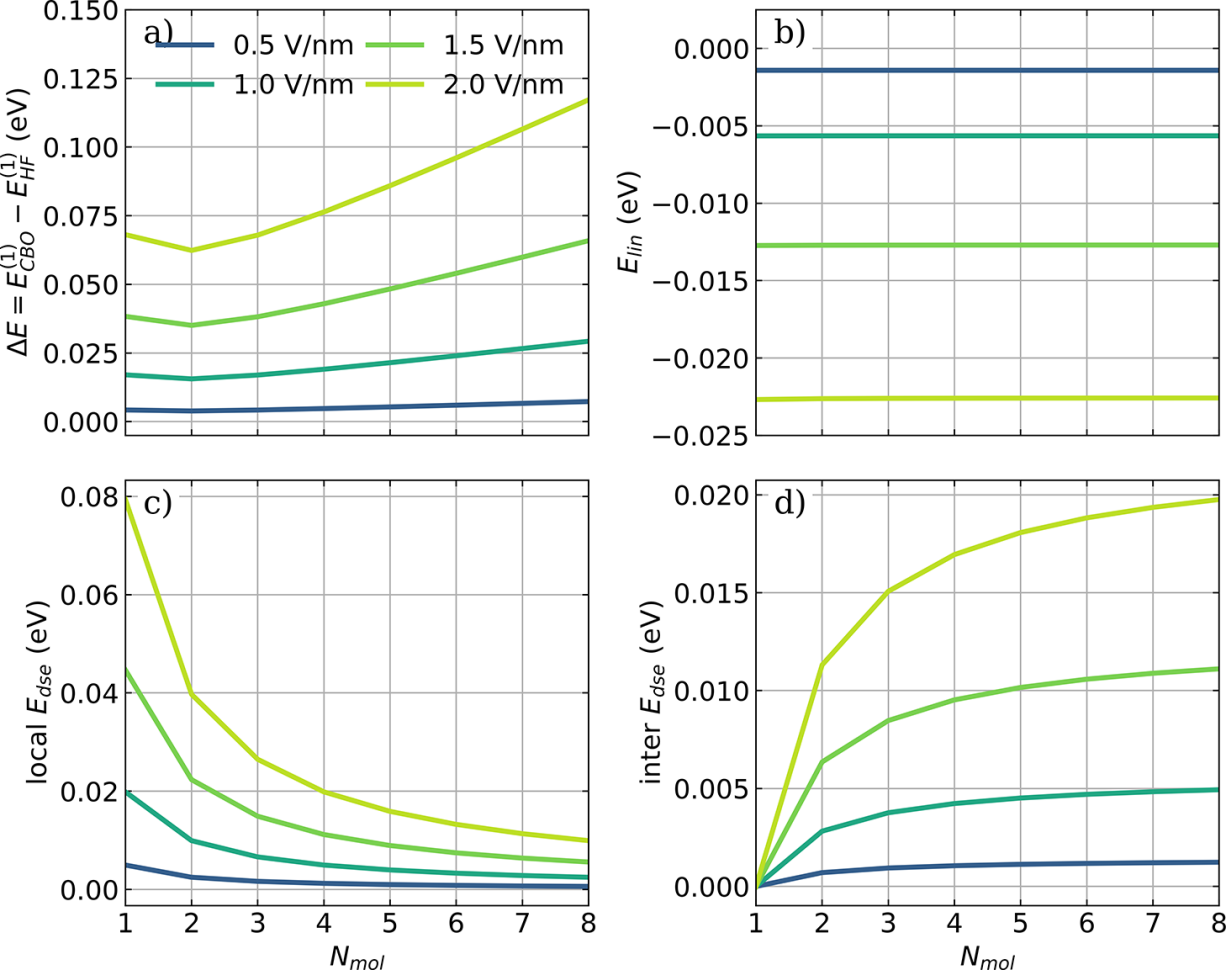
Influence of the cavity interaction on an individual HF molecule in *all-parallel* ensembles of different sizes and vacuum field strengths ϵ_*c*_. (a) The energy difference Δ*E* between *E*_*CBO*_^(1)^ and the field-free energy *E*_*HF*_^(1)^, (b) the local linear coupling contribution *E*_*lin*_, (c) the *local E*_*dse*_, and (d) the *inter E*_*dse*_ that is part of *E*_*dse*_^(2*J*)^ (for definition, see eq 12) as a function of *N*_*mol*_. The individual dipole moments are aligned with the cavity polarization axis, and a cavity frequency ω_*c*_ of 4467 cm^–1^ is used. The strength of the cavity field ϵ_*c*_ increases from 0.5 to 2.0 V nm^–1^ (color-coded). The coupling strength **λ**_*c*_ used is rescaled according to eq 16.

The energy difference Δ*E* between the individual molecular energy *E*_*CBO*_^(1)^ and *E*_*HF*_^(1)^ of the isolated HF molecule without cavity interaction is shown in Figure 3a. Note that *E*_*dis*_ is an ensemble quantity, but since each molecule in the ensemble is affected by the same potential, we include it in *E*_*CBO*_^(1)^. If a second molecule is included in the cavity, then the local *E*_*CBO*_^(1)^ decreases. As more and more HF molecules are added, the initial trend reverses, and *E*_*CBO*_^(1)^ increases almost linearly, following the linear behavior of *E*_*dis*_ shown in Figure 2b. Without *E*_*dis*_, *E*_*CBO*_^(1)^ converges to a finite nonzero value with *N*_*mol*_ increasing, as can be seen in Figure S4a in the Supporting Information. The local dipole field interaction *E*_*lin*_ converges to a constant value with increasing *N*_*mol*_, as shown in Figure 3b. This behavior is a direct consequence of fulfilling the zero-field condition for the entire *all-parallel* ensemble and cannot be generalized to every nuclear configuration. For this specific orientation, ensemble dipole moment **μ** increases linearly with the number of molecules, and thus, the displacement induced by the cavity leads to higher values of *q*_min_. This effect, in combination with the rescaled coupling **λ**_*c*_ leads to the constant value of *E*_*lin*_ that depends only on the coupling strength. On the contrary, the *local E*_*dse*_, visualized in Figure 3c, decays with  and approaches zero in the large *N*_*mol*_ limit. The intermolecular dipole–dipole energy (*inter E*_*dse*_) shown in Figure 3d is part of the *E*_*dse*_^(2*J*)^ term and arises as a result of the cavity-mediated interaction of the dipole moment of one molecule with all other molecules in the ensemble (for the definition, see eq 12). This energy contribution increases with an increasing number of molecules in the ensemble and approaches a constant, nonzero value following the behavior of 1 – . For the *all-parallel* configuration, the single molecule interacts with *N*_*mol*_ – 1 identical molecules via the DSE term, which is scaled by a factor of  (see eq 16). All of these results are clear indications that the nontrivial interplay of the collective photon displacement effects (*E*_*lin*_ in combination with *E*_*dis*_) and the cavity-mediated dipole–dipole interaction allow for local strong coupling to emerge.

In the following, we introduce a defect in perfectly aligned ensembles to further study the effect of anisotropic ensembles on a single molecule. We perform scans along the bond length of one HF molecule in fixed ensembles of different sizes for all three configurations *all-parallel*, *antiparallel*, and *defective*. The scan along the bond length is performed for the HF molecule highlighted in red in Figure 1. In the *defective* orientation, the fixed *N*_*mol*_ – 1 HF molecules point in the opposite direction to the perturbed molecule. For the resulting two-dimensional cavity potential energy surfaces (cPESs) spanned by the bond length coordinate and the photon displacement coordinate, the minimum energy path along the bond length is determined. This is equivalent to satisfying the zero transverse electric field condition (eq 14) for each nuclear configuration. The energy differences between these minimum energy paths and the field-free, one-dimensional potential energy surface (PES) for all three orientations are visualized in Figure 4. The corresponding energy contributions *E*_*lin*_, *E*_*dis*_, the *local E*_*dse*_, and the *inter E*_*dse*_ are shown in Figures S7–S9 in section S4 of the Supporting Information.

**Figure 4:**
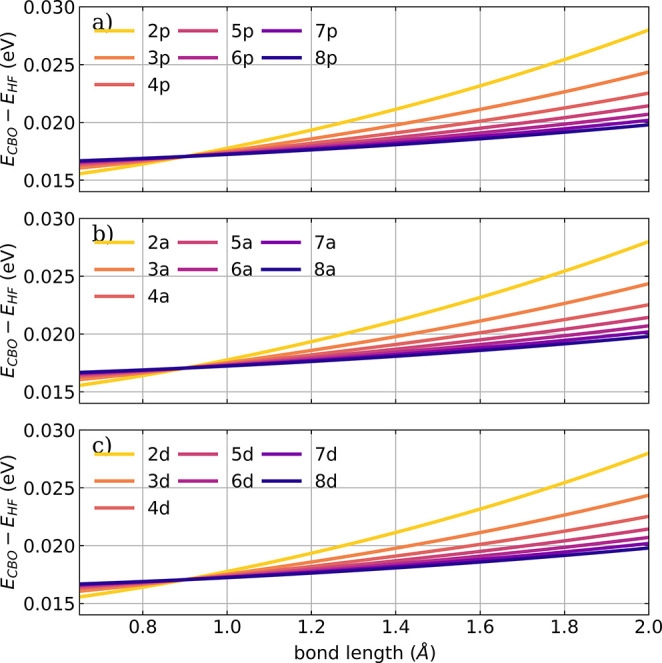
Cavity-induced energy change along the HF bond length for different ensemble sizes and configurations: (a) *all-parallel* configuration, (b) *antiparallel* configuration, and (c) *defective* configuration; for definitions, see Figure 1. A cavity frequency ω_*c*_ of 4467 cm^–1^ is used, and the strength of the cavity field ϵ_*c*_ is set to 1.5 V nm^–1^. The coupling strength **λ**_*c*_ used is rescaled according to eq 16 and the number of molecules in the ensemble is color-coded.

The observed change in the energetics of the dissociation path due to the cavity interaction is small (see Figure 4), and interestingly, it is the same for all three ensemble configurations. However, the effect is not negligible, and the cavity interaction shifts the ensemble cPESs to higher energies compared to the cavity-free PES. With increasing bond length, the ensemble dipole moment becomes larger and consequently the upshift mediated by the cavity interaction increases. When comparing the different sizes of the ensemble, there is a decreasing effect on the change in the energy of the ensemble with increasing *N*_*mol*_. As a second effect, the energy change is less and less dependent on the bond length. It seems to converge to a nonzero finite value, which is constant with respect to the bond length. A closer look at the individual contributions *E*_*lin*_, *E*_*dis*_, the *local E*_*dse*_, and the *inter E*_*dse*_ all shown in Figures S7–S9 in section S4 of the Supporting Information can explain these trends. The general shape of the cavity-induced energy change shown in Figure 4 is determined by the *local E*_*dse*_. With an increasing number of molecules in the cavity, this contribution becomes dominated by the fixed ensemble of *N*_*mol*_ – 1 molecules and therefore constant. The other three contributions, *E*_*lin*_, *E*_*dis*_, and the *inter E*_*dse*_, are quite large and depend both on the size of the ensemble and the bond length. However, when they are summed, they almost completely cancel each other out, leaving only a negligible energy contribution. Since the *local E*_*dse*_ is the same for all three orientations and dominates the energy change, all three ensemble configurations show the same behavior. It should be noted that by imposing the zero transverse electric field condition along the complete dissociation path, we assume that the whole ensemble coupled to the cavity is in the electronic–photonic ground state. The behavior discussed above may be different if this assumption no longer holds, for example, if the system is coupled to a thermal bath.

In the last part of this work, we focus on the local perspective of the dissociating HF molecule. Its field-free PES and the change in the local energy induced by the cavity interaction in the presence of different ensembles are shown in Figure 5. Additional figures can be found in section S4 of the Supporting Information, showing the underlying energy components: *E*_*lin*_ in Figure S10, *E*_*dis*_ in Figure S11, the *local E*_*dse*_ in Figure S12, and the *inter E*_*dse*_ in Figure S13. The last two are calculated from the perspective of the dissociating molecule.

**Figure 5:**
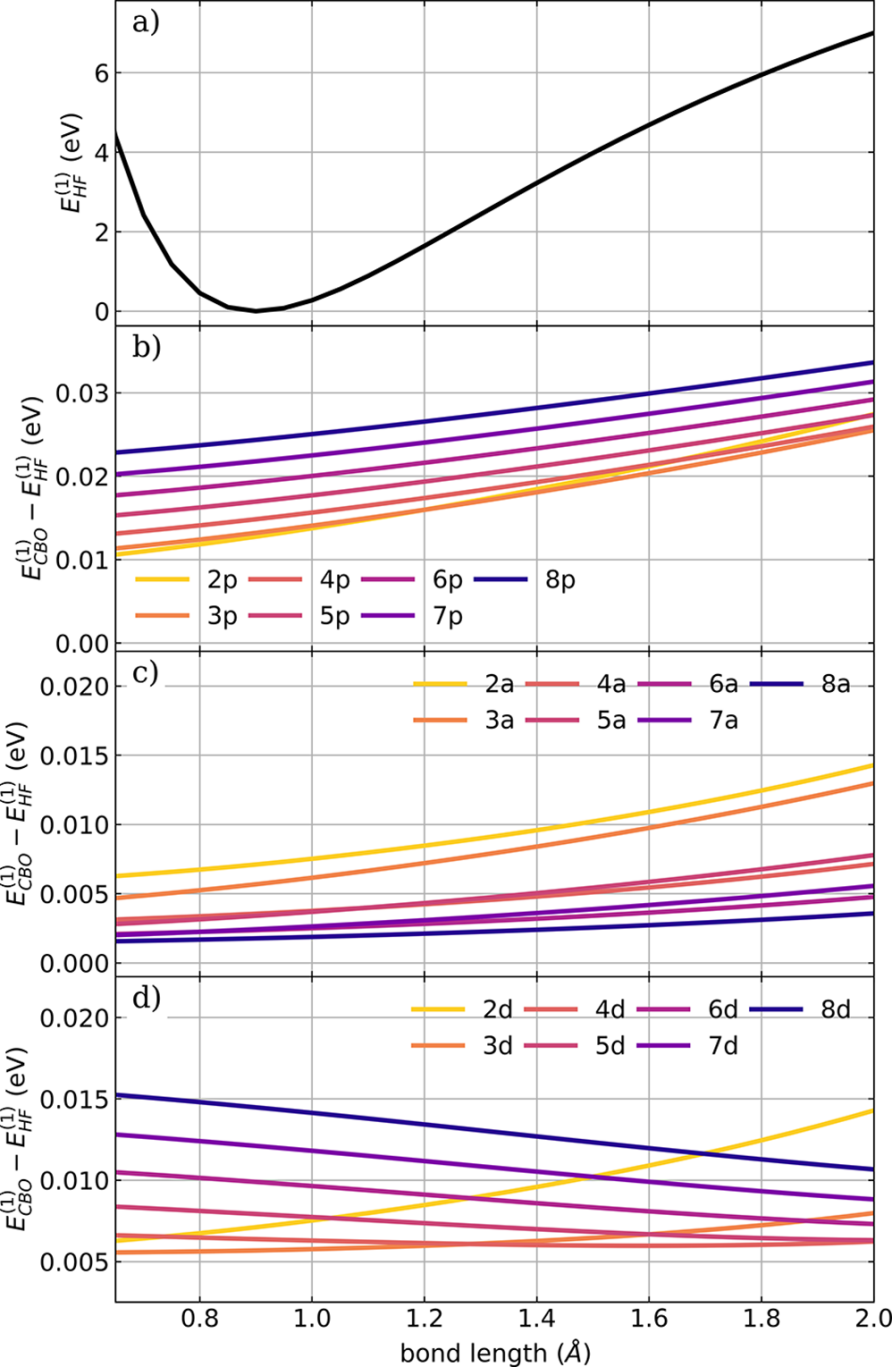
(a) Field-free PES of a single dissociating HF molecule. Cavity-induced energy change on the individual dissociating HF molecule in ensembles of different size in (b) the *all-parallel* configuration, (c) *antiparallel* configuration, and (d) the *defective* configuration; for definitions, see Figure 1. The energy change is calculated as the difference between the locale energy *E*_*CBO*_^(1)^ and the field-free energy *E*_*HF*_^(1)^. A cavity frequency ω_*c*_ of 4467 cm^–1^ is used, and the strength of the cavity field ϵ_*c*_ is set to 1.5 V nm^–1^. The coupling strength **λ**_*c*_ used is rescaled according to eq 16, and the number of molecules in the ensemble is color-coded.

The most striking difference from the perspective of the ensemble is that at the local level of the dissociating HF molecule, the three configurations *all-parallel* (Figure 5b), *antiparallel* (Figure 5c) and *defective* (Figure 5d) can be distinguished in cavity-induced energy changes. In all three situations, the cavity-induced changes depend on *N*_*mol*_ as well as on the length of the bond. In the *all-parallel* case, shown in Figure 5b, the energy change increases with the bond length as well as with the number of molecules. Note that the coupling is rescaled by  to keep the collective Rabi-splitting constant. However, the effect on the individual molecules grows with the number of molecules in the ensemble, even though the single-molecule coupling strength λ_0_ becomes smaller. We thus conclude that collective coupling induces locally strong effects, as has been reported for excited states in ESC previously.^51^ The local contribution *E*_*lin*_, which converges to a finite value, is not large enough to compensate for *E*_*dis*_, which grows linearly with the size of the ensemble (see Figures S10a and S11a). The resulting upshift in energy is further amplified since *inter E*_*dse*_ is also positive due to the *all-parallel* configuration and grows with  (see Figure S13a). Also in the *antiparallel* case (Figure 5c), the local energy change due to the cavity interaction increases with increasing bond length. However, the cavity-induced energy change is generally smaller than that for the *all-parallel* configuration and becomes significantly smaller with increasing *N*_*mol*_. The curves, shown in Figure 5c, have a pairwise structure, where each ensemble with an even value of *N*_*mol*_ is very close in energy to the ensemble with *N*_*mol*_ + 1 molecules. The case of odd and even numbers of molecules in the *antiparallel* configuration creates two different situations from a single-molecule perspective. For even *N*_*mol*_ the whole ensemble reduces to an effective antiparallel bimolecular case, and the situation of odd *N*_*mol*_ is equivalent to the single-molecule case where both are scaled down by . Therefore, *E*_*lin*_ and *E*_*dis*_ become smaller with increasing *N*_*mol*_ for odd and even *N*_*mol*_ (see Figures S10b and S11b). Furthermore, for even *N*_*mol*_ ensembles, there is a small negative contribution from *inter E*_*dse*_ (see Figure S13b). In contrast to the *all-parallel* and *antiparallel* configurations, the ensembles in the *defective* orientation show clearly different trends. Similarly to the *all-parallel* case, Δ*E* increases as *N*_*mol*_ increases for a fixed nuclear configuration, but for *N*_*mol*_ > 3, it simultaneously decreases along the dissociation path. This change in behavior is caused by the interplay of local *E*_*lin*_, ensemble quantity *E*_*dis*_, and cavity-mediated dipole–dipole interaction. These three contributions are shown in Figures S10c, S11c, and S12c). For ensembles with *N*_*mol*_ < 4 the total dipole moment changes sign along the dissociation path, while for a larger ensemble it is negative for the whole pathway. The local dipole moment of the dissociating molecule is positive in the studied configuration, which leads to a positive contribution of the local *E*_*lin*_ for *N*_*mol*_ > 3 and a change in sign for *E*_*lin*_ in smaller ensembles. The *inter E*_*dse*_ (see Figure S13c) acting on the dissociating molecule is always negative and is increasingly relevant for increasing bond length. Consequently, the cavity-induced energy shift from the local perspective decreases for the *defective* configuration along the dissociation pathway. In summary, for all three configurations studied, the interaction of the cavity with the molecular ensembles modifies the local energy landscape of the individual molecule. These changes are strongly dependent on the ensemble properties (size and orientation of the components) and are due to the interplay of the displacement field effects and the cavity-induced polarization.

In conclusion, we have established an *ab initio* Hartree–Fock ansatz in the framework of the cavity Born–Oppenheimer approximation, capable of describing the electronic ground state of molecular systems coupled to an optical cavity. We have applied the CBO-HF ansatz to study the collective effects in small ensembles of diatomic hydrogen fluoride (HF) molecules in an optical cavity. The detailed analysis of the cavity-induced energy changes for the whole ensemble and individual molecules shows that the self-consistent treatment and the full dipole self-energy operator are crucial to capture relevant aspects for the description of a strongly coupled molecular ensemble and its chemical properties. The DSE terms are essential to describe cavity-induced polarization at the level of individual molecules as well as for the whole ensemble. The observed interplay of displacement field effects and the cavity-induced polarization enables energy changes for the individual molecule because of collective coupling to an optical cavity. Consistent with our previous work^42^ we could identify a macroscopically induced microscopic polarization mechanism based on intermolecular dipole–dipole interactions. Although we have only studied the system in the electronic–photonic ground state, we see indications that thermal fluctuations may play a decisive role in polaritonic chemistry, in line with our previous work.^42^ Due to the nature of the intermolecular dipole–dipole interactions, a local change/fluctuation in the dipole moment and/or polarizability could affect the whole ensemble. A prepolarization of an ensemble with a static electric field, for example, should lead to an observable effect in experiment. Another interesting topic for further study is the interplay of this self-consistent polarization mechanism with vibrational or electronic resonances.

The derivation of the CBO-HF equations demonstrates which molecular properties are important for the DSE term and the couplings it introduces: molecular dipole moments are important for intermolecular interactions, while the combination of dipole moments, quadrupole moments, and transition dipole moments are important on an intramolecular level. The CBO-HF ansatz and the underlying CBOA formulation offers a suitable framework to derive post-Hartree–Fock methods, such as configuration interaction or coupled cluster, or potential self-consistent embedding schemes^69^ for molecules under VSC or even ESC.^26^ It may also provide potential energy surfaces that can be used for *ab initio* semiclassical dynamics or nuclear-photonic quantum dynamics simulations of molecular ensembles.

## Data Availability

All data underlying this study are available from the corresponding author upon reasonable request

## Supporting Information Available

Details of the derivation of the CBO-HF energy contribution, discussion of the single-molecule case and additional figures for the ensembles of HF molecules in an optical cavity (PDF)
