# Supplementary material for: Cavity Born–Oppenheimer Hartree–Fock Ansatz: Light–Matter Properties of Strongly Coupled Molecular Ensembles
Source: J Phys Chem Lett. 2023 Aug 31;14(36):8024–33. doi: 10.1021/acs.jpclett.3c01842 (PMC10510432; doi:10.1021/acs.jpclett.3c01842)
Supplement: Supplementary file 1 — jz3c01842_si_001.pdf [file jz3c01842_si_001.pdf]

Supporting Information: Cavity-Born-Oppenheimer Hartree-Fock Ansatz: Light-Matter Properties of Strongly Coupled Molecular Ensembles

Thomas Schnappinger,[†] Dominik Sidler,[‡,¶] Michael Ruggenthaler,[‡,¶] Angel Rubio,[‡,¶,§,‖] and Markus Kowalewski[*,†]

†*Department of Physics, Stockholm University, AlbaNova University Center, SE-106 91 Stockholm, Sweden*

‡*Max Planck Institute for the Structure and Dynamics of Matter and Center for Free-Electron Laser Science, Luruper Chaussee 149, 22761 Hamburg, Germany*

¶*The Hamburg Center for Ultrafast Imaging, Luruper Chaussee 149, 22761 Hamburg, Germany*

§*Center for Computational Quantum Physics, Flatiron Institute, 162 5th Avenue, New York, NY 10010, USA*

‖*Nano-Bio Spectroscopy Group, University of the Basque Country (UPV/EHU), 20018 San Sebastián, Spain*

E-mail: markus.kowalewski@fysik.su.se

Contents

S1 Derivation of the CBO-HF matrix elements **3**

S2 Single HF molecule in an optical cavity **8**

S3 Fixed ensembles of HF in an optical cavity **12**

S4 Scanned ensembles of HF molecules in an optical cavity **16**

References **24**

S1 Derivation of the CBO-HF matrix elements

In this section we provide a detailed derivation of the new matrix elements of the cavity Born-Oppenheimer Hartree-Fock (CBO-HF) ansatz. The Pauli-Fierz Hamiltonian for a single-mode cavity, in the length gauge and within the dipole approximation, and the cavity Born-Oppenheimer approximation (CBOA) has the form:[1–7]

$$\hat{H}_{CBO} = \hat{H}_{el} + \frac{1}{2}\omega_c^2 q_c^2 - \omega_c q_c \left(\boldsymbol{\lambda}_c \cdot \hat{\boldsymbol{\mu}}\right) + \frac{1}{2}\left(\boldsymbol{\lambda}_c \cdot \hat{\boldsymbol{\mu}}\right)^2 , \tag{S1}$$

where

$$\hat{\boldsymbol{\mu}} = \hat{\boldsymbol{\mu}}_{el} + \boldsymbol{\mu}_{Nuc} = -\sum_{i=1}^{N_{el}} \hat{\boldsymbol{r}}_i + \sum_{A=1}^{N_{Nuc}} Z_A \boldsymbol{R}_A , \tag{S2}$$

represents the molecular dipole operator, which is defined by the operators of the N_{el} electron coordinates $\hat{\boldsymbol{r}}$, the classic coordinates $\boldsymbol{R}$ of the N_{Nuc} nuclei and the nuclear charge Z. Using $\hat{H}_{CBO}$ and a Slater determinant Ψ, the CBO-HF energy expectation value E_{CBO} can be determined using the standard self-consistent field (SCF) procedure:[8]

$$\left\langle E_{CBO} \right\rangle = \left\langle \Psi \middle| \hat{H}_{el} - \omega_c q_c \left(\boldsymbol{\lambda}_c \cdot \hat{\boldsymbol{\mu}}\right) + \frac{1}{2}\left(\boldsymbol{\lambda}_c \cdot \hat{\boldsymbol{\mu}}\right)^2 \middle| \Psi \right\rangle + \frac{1}{2}\omega_c^2 q_c^2 \tag{S3}$$

with the Slater determinant Ψ defined as follows:

$$\Psi(\boldsymbol{\tau}_1, \boldsymbol{\tau}_2, \ldots, \boldsymbol{\tau}_N) = \frac{1}{\sqrt{N_{el}!}} |\varphi_1, \varphi_2, \ldots \varphi_i\rangle. \tag{S4}$$

The resulting energy expectation E_{CBO} consists of four energy contributions:

$$E_{CBO} = E_{el} + E_{lin} + E_{dse} + E_{dis} \quad \text{with} \quad E_{dis} = \frac{1}{2}\omega_c^2 q_c^2 \tag{S5}$$

The terms E_{lin} and E_{dse} represent the liner light-matter coupling via the molecular dipole moment and the dipole self energy contribution due to self-polarization, respectively. Both can be extended to one- and two-electron contributions. In the following, we express them in terms of matrix elements of one- and two-electron operators between a N_{el}-electron Slater determinant, following the standard rules for Hartree-Fock matrix elements.[8] The energy contribution E_{lin} is formulated as modified dipole moment integrals and a parametric nuclear contribution:

$$\begin{aligned} E_{lin} &= -\omega_c q_c \langle \Psi | \boldsymbol{\lambda}_c \cdot \hat{\boldsymbol{\mu}}_{\boldsymbol{el}} | \Psi \rangle - \omega_c q_c \left(\boldsymbol{\lambda}_c \cdot \boldsymbol{\mu}_{Nuc} \right) = \omega_c q_c \sum_{i=1}^{N_{el}} \langle \Psi | \boldsymbol{\lambda}_c \cdot \hat{\boldsymbol{r}}_i | \Psi \rangle - \omega_c q_c \left(\boldsymbol{\lambda}_c \cdot \boldsymbol{\mu}_{Nuc} \right) \\ &= \omega_c q_c N_{el} \langle \Psi | \boldsymbol{\lambda}_c \cdot \hat{\boldsymbol{r}} | \Psi \rangle - \omega_c q_c \left(\boldsymbol{\lambda}_c \cdot \boldsymbol{\mu}_{Nuc} \right) = \omega_c q_c \sum_{i=1}^{N_{oc}} \langle \varphi_i | \boldsymbol{\lambda}_c \cdot \hat{\boldsymbol{r}} | \varphi_i \rangle - \omega_c q_c \left(\boldsymbol{\lambda}_c \cdot \boldsymbol{\mu}_{Nuc} \right) \end{aligned} \tag{S6}$$

Before deriving E_{dse} in terms of matrix elements, we decompose the operator describing the dipole self-energy (DSE) into a purely electronic operator $\hat{H}_{dse}^{(el)}$, a mixed electron-nuclear operator $\hat{H}_{dse}^{(e\text{-}n)}$, and a parametric nuclear energy contribution $E_{dse}^{(nuc)}$.

$$\begin{aligned} \frac{1}{2} \left(\boldsymbol{\lambda}_c \cdot \hat{\boldsymbol{\mu}} \right)^2 =& \frac{1}{2} \left(- \sum_{i=1}^{N_{el}} \boldsymbol{\lambda}_c \cdot \hat{\boldsymbol{r}}_i + \boldsymbol{\lambda}_c \cdot \boldsymbol{\mu}_{Nuc} \right)^2 \\ =& \frac{1}{2} \left(\sum_{i=1}^{N_{el}} \boldsymbol{\lambda}_c \cdot \hat{\boldsymbol{r}}_i \right)^2 - \left(\sum_{i=1}^{N_{el}} \boldsymbol{\lambda}_c \cdot \hat{\boldsymbol{r}}_i \right) \left(\boldsymbol{\lambda}_c \cdot \boldsymbol{\mu}_{Nuc} \right) + \frac{1}{2} \left(\boldsymbol{\lambda}_c \cdot \boldsymbol{\mu}_{Nuc} \right)^2 \\ =& \frac{1}{2} \left(\sum_{i=1}^{N_{el}} \boldsymbol{\lambda}_c \cdot \hat{\boldsymbol{r}}_i \right)^2 - \left(\sum_{i=1}^{N_{el}} \boldsymbol{\lambda}_c \cdot \hat{\boldsymbol{r}}_i \right) \left(\boldsymbol{\lambda}_c \cdot \boldsymbol{\mu}_{Nuc} \right) + E_{dse}^{(nuc)} \\ =& \hat{H}_{dse}^{(el)} + \hat{H}_{dse}^{(e\text{-}n)} + E_{dse}^{(nuc)} \end{aligned} \tag{S7}$$

The purely electronic contribution $E_{dse}^{(el)}$ is decomposed into the one-electron part $E_{dse}^{(1)}$ and the two-electron part $E_{dse}^{(2)}$:

$$
\begin{aligned}
E_{dse}^{(el)} &= \langle \Psi | \hat{H}_{dse}^{(el)} | \Psi \rangle = \frac{1}{2} \langle \Psi | \left(-\sum_{i=1}^{N_{el}} \boldsymbol{\lambda}_c \cdot \hat{\boldsymbol{r}}_i \right)^2 | \Psi \rangle = \frac{1}{2} \langle \Psi | \left(-\sum_{i=1}^{N_{el}} \boldsymbol{\lambda}_c \cdot \hat{\boldsymbol{r}}_i \right) \left(-\sum_{j=1}^{N_{el}} \boldsymbol{\lambda}_c \cdot \hat{\boldsymbol{r}}_j \right) | \Psi \rangle \\
&= \frac{1}{2} \langle \Psi | \sum_{i=1}^{N_{el}} \left(\boldsymbol{\lambda}_c \cdot \hat{\boldsymbol{r}}_i \right)^2 + \sum_{i=1}^{N_{el}} \sum_{j \neq i}^{N_{el}} \left(\boldsymbol{\lambda}_c \cdot \hat{\boldsymbol{r}}_i \right) \left(\boldsymbol{\lambda}_c \cdot \hat{\boldsymbol{r}}_j \right) | \Psi \rangle \\
&= \frac{1}{2} \sum_{i=1}^{N_{el}} \langle \Psi | \left(\boldsymbol{\lambda}_c \cdot \hat{\boldsymbol{r}}_i \right)^2 | \Psi \rangle + \frac{1}{2} \sum_{i=1}^{N_{el}} \sum_{j \neq i}^{N_{el}} \langle \Psi | \left(\boldsymbol{\lambda}_c \cdot \hat{\boldsymbol{r}}_i \right) \left(\boldsymbol{\lambda}_c \cdot \hat{\boldsymbol{r}}_j \right) | \Psi \rangle = E_{dse}^{(1e)} + E_{dse}^{(2e)}
\end{aligned}
\tag{S8}
$$

The energy contribution $E_{dse}^{(1e)}$ can be formulated as modified quadrupole moment integrals:

$$
E_{dse}^{(1e)} = \frac{1}{2} \sum_{i=1}^{N_{el}} \langle \Psi | \left(\boldsymbol{\lambda}_c \cdot \hat{\boldsymbol{r}}_i \right)^2 | \Psi \rangle = \frac{1}{2} N_{el} \langle \Psi | \left(\boldsymbol{\lambda}_c \cdot \hat{\boldsymbol{r}} \right)^2 | \Psi = \frac{1}{2} \sum_{i=1}^{N_{oc}} \langle \varphi_i | \left(\boldsymbol{\lambda}_c \cdot \hat{\boldsymbol{r}} \right)^2 | \varphi_i \rangle \tag{S9}
$$

Since $E_{dse}^{(2e)}$ connects the position operators of two electrons i and j, its transformation into Hartree-Fock matrix elements, follows a similar logic as the derivation of the Coulomb interaction in a regular Hartree-Fock ansatz:[8]

$$
\begin{aligned}
E_{dse}^{(2e)} &= \frac{1}{2} \sum_{i=1}^{N_{el}} \sum_{j \neq i}^{N_{el}} \langle \Psi | \left(\boldsymbol{\lambda}_c \cdot \hat{\boldsymbol{r}}_i \right) \left(\boldsymbol{\lambda}_c \cdot \hat{\boldsymbol{r}}_j \right) | \Psi \rangle = \frac{1}{2} N_{el} \left(N_{el} - 1 \right) \langle \Psi | \left(\boldsymbol{\lambda}_c \cdot \hat{\boldsymbol{r}}_1 \right) \left(\boldsymbol{\lambda}_c \cdot \hat{\boldsymbol{r}}_2 \right) | \Psi \rangle \\
&= \frac{1}{2} \sum_{i=1}^{N_{oc}} \sum_{j \neq i}^{N_{oc}} \langle \varphi_i | \left(\boldsymbol{\lambda}_c \cdot \hat{\boldsymbol{r}} \right) | \varphi_i \rangle \langle \varphi_j | \left(\boldsymbol{\lambda}_c \cdot \hat{\boldsymbol{r}} \right) | \varphi_j \rangle - \langle \varphi_i | \left(\boldsymbol{\lambda}_c \cdot \hat{\boldsymbol{r}} \right) | \varphi_j \rangle \langle \varphi_j | \left(\boldsymbol{\lambda}_c \cdot \hat{\boldsymbol{r}} \right) | \varphi_i \rangle
\end{aligned}
\tag{S10}
$$

Since the case $i = j$ is equal to zero, the restriction on the summation can be removed:

$$
\begin{aligned}
E_{dse}^{(2e)} =& \frac{1}{2}\sum_{i=1}^{N_{oc}}\sum_{j=1}^{N_{oc}} \langle\varphi_i|\left(\boldsymbol{\lambda}_c\cdot\hat{\boldsymbol{r}}\right)|\varphi_i\rangle\langle\varphi_j|\left(\boldsymbol{\lambda}_c\cdot\hat{\boldsymbol{r}}\right)|\varphi_j\rangle - \langle\varphi_i|\left(\boldsymbol{\lambda}_c\cdot\hat{\boldsymbol{r}}\right)|\varphi_j\rangle\langle\varphi_j|\left(\boldsymbol{\lambda}_c\cdot\hat{\boldsymbol{r}}\right)|\varphi_i\rangle \\
=& \frac{1}{2}\sum_{i=1}^{N_{oc}}\sum_{j=1}^{N_{oc}} \langle\varphi_i|\left(\boldsymbol{\lambda}_c\cdot\hat{\boldsymbol{r}}\right)|\varphi_i\rangle\langle\varphi_j|\left(\boldsymbol{\lambda}_c\cdot\hat{\boldsymbol{r}}\right)|\varphi_j\rangle - \left|\langle\varphi_i|\left(\boldsymbol{\lambda}_c\cdot\hat{\boldsymbol{r}}\right)|\varphi_j\rangle\right|^2 \\
=& \frac{1}{2}E_{dse}^{(2J)} + \frac{1}{2}E_{dse}^{(2K)}
\end{aligned}
\tag{S11}
$$

The resulting two parts are a Coulomb-like dipole-dipole interaction component $E_{dse}^{(2J)}$ and an exchange-like component $E_{dse}^{(2K)}$, which are calculated via modified dipole moment integrals. The mixed electron-nuclear operator $\hat{H}_{dse}^{(e\text{-}n)}$ leads to the energy contribution $E_{dse}^{(e\text{-}n)}$, which is formulated as a product of modified dipole moment integrals and a parametric nuclear contribution:

$$
\begin{aligned}
E_{dse}^{(e\text{-}n)} &= \langle\Psi|\hat{H}_{dse}^{(e\text{-}n)}|\Psi\rangle = -\left(\boldsymbol{\lambda}_c\cdot\boldsymbol{\mu}_{Nuc}\right)\sum_{i=1}^{N_{el}}\langle\Psi|\boldsymbol{\lambda}_c\cdot\hat{\boldsymbol{r}}_i|\Psi\rangle \\
&= \left(\boldsymbol{\lambda}_c\cdot\boldsymbol{\mu}_{Nuc}\right)N_{el}\langle\Psi|\boldsymbol{\lambda}_c\cdot\hat{\boldsymbol{r}}|\Psi\rangle = \left(\boldsymbol{\lambda}_c\cdot\boldsymbol{\mu}_{Nuc}\right)\sum_{i=1}^{N_{oc}}\langle\varphi_i|\boldsymbol{\lambda}_c\cdot\hat{\boldsymbol{r}}|\varphi_i\rangle
\end{aligned}
\tag{S12}
$$

The contribution $E_{dse}^{(nuc)}$ depends only on the nuclear part of the dipole moment and is added as a scalar quantity to E_{CBO}.

In the last part of this section we will briefly discuss the underlying modified dipole moment integrals and modified quadrupole moment integrals. For modified dipole moments, the regular integrals are simply multiplied by the corresponding Cartesian component of the coupling strength $\boldsymbol{\lambda}_c$ and then summed over the three Cartesian coordinates.

$$
\begin{aligned}
\langle\varphi_i|\left(\boldsymbol{\lambda}_c\cdot\hat{\boldsymbol{r}}\right)|\varphi_i\rangle &= \langle\varphi_i|\lambda_x\hat{r}_x + \lambda_y\hat{r}_y + \lambda_z\hat{r}_z|\varphi_i\rangle \\
&= \lambda_x\langle\varphi_i|\hat{r}_x|\varphi_i\rangle + \lambda_y\langle\varphi_i|\hat{r}_y|\varphi_i\rangle + \lambda_z\langle\varphi_i|\hat{r}_z|\varphi_i\rangle
\end{aligned}
\tag{S13}
$$

For the modified quadrupole moment integrals the situation is slightly more complicated. All quadrupole moment tensor elements are multiplied with the two corresponding Cartesian components of the coupling strength $\boldsymbol{\lambda}_c$ and then summed over all elements.

$$
\begin{aligned}
\left\langle\varphi_i\right| (\boldsymbol{\lambda}_c \cdot \hat{\boldsymbol{r}})^2 \left|\varphi_i\right\rangle =& \left\langle\varphi_i\right| (\lambda_x \hat{r}_x + \lambda_y \hat{r}_y + \lambda_z \hat{r}_z)^2 \left|\varphi_i\right\rangle \\
=& \left\langle\varphi_i\middle|\lambda_x^2 \hat{r}_x^2 + \lambda_y^2 \hat{r}_y^2 + \lambda_z^2 \hat{r}_z^2 + 2\lambda_x\lambda_y \hat{r}_x \hat{r}_y + 2\lambda_x\lambda_z \hat{r}_x \hat{r}_z + 2\lambda_y\lambda_z \hat{r}_y \hat{r}_z\middle|\varphi_i\right\rangle \\
=& \lambda_x^2 \left\langle\varphi_i\middle|\hat{r}_x^2\middle|\varphi_i\right\rangle + \lambda_y^2 \left\langle\varphi_i\middle|\hat{r}_y^2\middle|\varphi_i\right\rangle + \lambda_z^2 \left\langle\varphi_i\middle|\hat{r}_z^2\middle|\varphi_i\right\rangle + 2\lambda_x\lambda_y \left\langle\varphi_i\middle|\hat{r}_x \hat{r}_y\middle|\varphi_i\right\rangle \\
&+ 2\lambda_x\lambda_z \left\langle\varphi_i\middle|\hat{r}_x \hat{r}_z\middle|\varphi_i\right\rangle + 2\lambda_y\lambda_z \left\langle\varphi_i\middle|\hat{r}_y \hat{r}_z\middle|\varphi_i\right\rangle
\end{aligned}
\tag{S14}
$$

S2 Single HF molecule in an optical cavity

The energy change of a single HF molecule induced by the interaction with the cavity, as well as the underlying energy components, are visualized in Fig. S1. For this purpose q_c is scanned for different cavity field strengths and a fixed nuclear configuration. The molecular dipole moment $\boldsymbol{\mu}$ is aligned with the polarization axis of the cavity.

For the coupling strengths studied, the cavity potential energy surfaces (cPESs) defined by E_{CBO} (Fig. S1 a)) are basically shifted versions of the harmonic potential E_{dis}, for definition, see Eq. S5. With increasing ϵ_c the minimum of the cPES defined by q_{min} is shifted to higher values of q_c and simultaneously to higher energies. The shift in q_c is due to E_{lin} describing the energy induced by the coupling between the molecule and the photon displacement field. As shown in Fig. S1 b) E_{lin} is a nearly linear function of q_c with a zero crossing at $q_c = 0.0$ and a slope that increases with ϵ_c. The shift of E_{CBO} towards higher energies is caused by E_{dse}. This contribution, see Fig. S1 c), is for the conditions studied nearly constant when changing q_c and its value increases with increasing ϵ_c. To get an impression of how the quantities just discussed (E_{CBO}, E_{lin}, and E_{dse}) depend on the orientation of the molecular dipole moment, the angle ϕ between $\boldsymbol{\mu}$ and the unit vector pointing along the cavity mode polarization axis is scanned. The energy values obtained for optimized q_{min} are visualized in Fig. S2

The cPESs shown in Fig. S2 a) have a clear minimum for $\phi = 90°$, which corresponds to $\boldsymbol{\mu}$ being orthogonal to the cavity polarization axes. Parallel orientation (0°) and antiparallel orientation (180°) are maxima/transition states along the rotation coordinate defined by ϕ, although the interaction with the cavity is maximal (Fig. S2 b and c)) for these configurations. For $\phi = 90°$ there is no direct dipole cavity interaction, $E_{lin} = 0.0\,\text{eV}$ see Fig. S2 c) and $E_{dis} = 0.0\,\text{eV}$ see Fig. S2 b). However, the energy difference between cPESs and cavity-free potential energy surface (PES) is not zero for $\phi = 90°$. This is due to E_{dse}, which is not zero in this orientation, as shown in Fig. S2 d)). In Fig. S3 a further decomposition of E_{DSE} is shown. The overall highest contribution is $E_{dse}^{(1e)}$ and both $E_{dse}^{(1e)}$ and $E_{dse}^{(2K)}$ are non-zero for

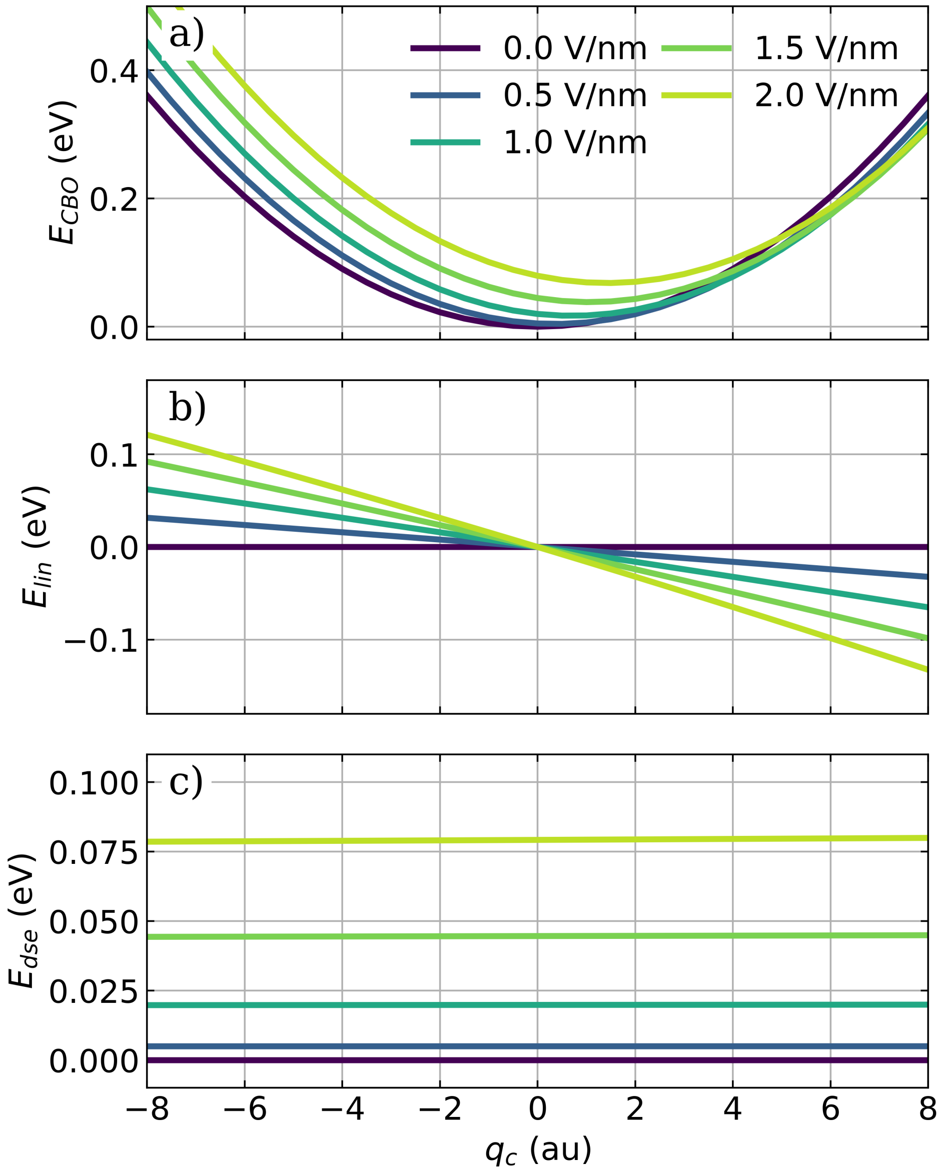

Figure S1: Scan along the photon displacement coordinate q_c for a fixed single HF molecule of the total energy E_{CBO} (a)), the linear energy contribution E_{lin} (b)) and the DSE part E_{dse} (c)). All scans were performed with the molecular dipole moment $\boldsymbol{\mu}$ aligned with the cavity polarization axis, a cavity frequency ω_c of $4467\,\mathrm{cm}^{-1}$ and the cavity field strengths ϵ_c is increased from $0.0\,\mathrm{V\,nm}^{-1}$ to $2.0\,\mathrm{V\,nm}^{-1}$ (color-coded).

all possible orientations. Only $E_{dse}^{(2J)}$ is zero for $\phi = 90°$.

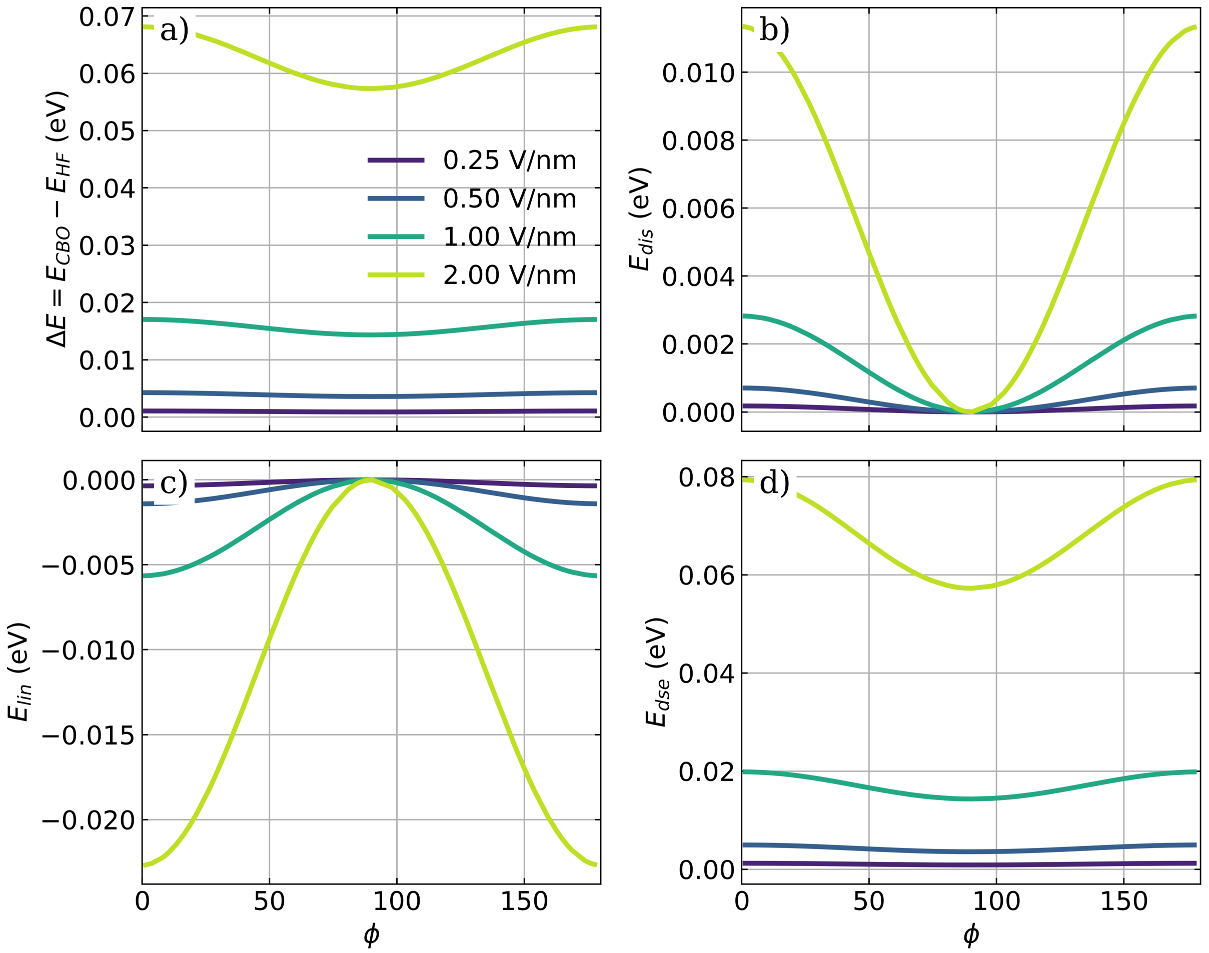

Figure S2: Scan along the angle ϕ, defined between $\boldsymbol{\mu}$ of a single HF and the polarization axis $\boldsymbol{e}$. a) ΔE between the rotational cPESs and the cavity-free PES, b) E_{dis}, c) E_{lin}, and d) E_{dse} for optimized q_{min}. All scans were performed with a cavity frequency ω_c of $4467\,\mathrm{cm}^{-1}$ and the cavity field strengths ϵ_c is increased from $0.25\,\mathrm{V\,nm}^{-1}$ to $2.0\,\mathrm{V\,nm}^{-1}$ (color-coded).

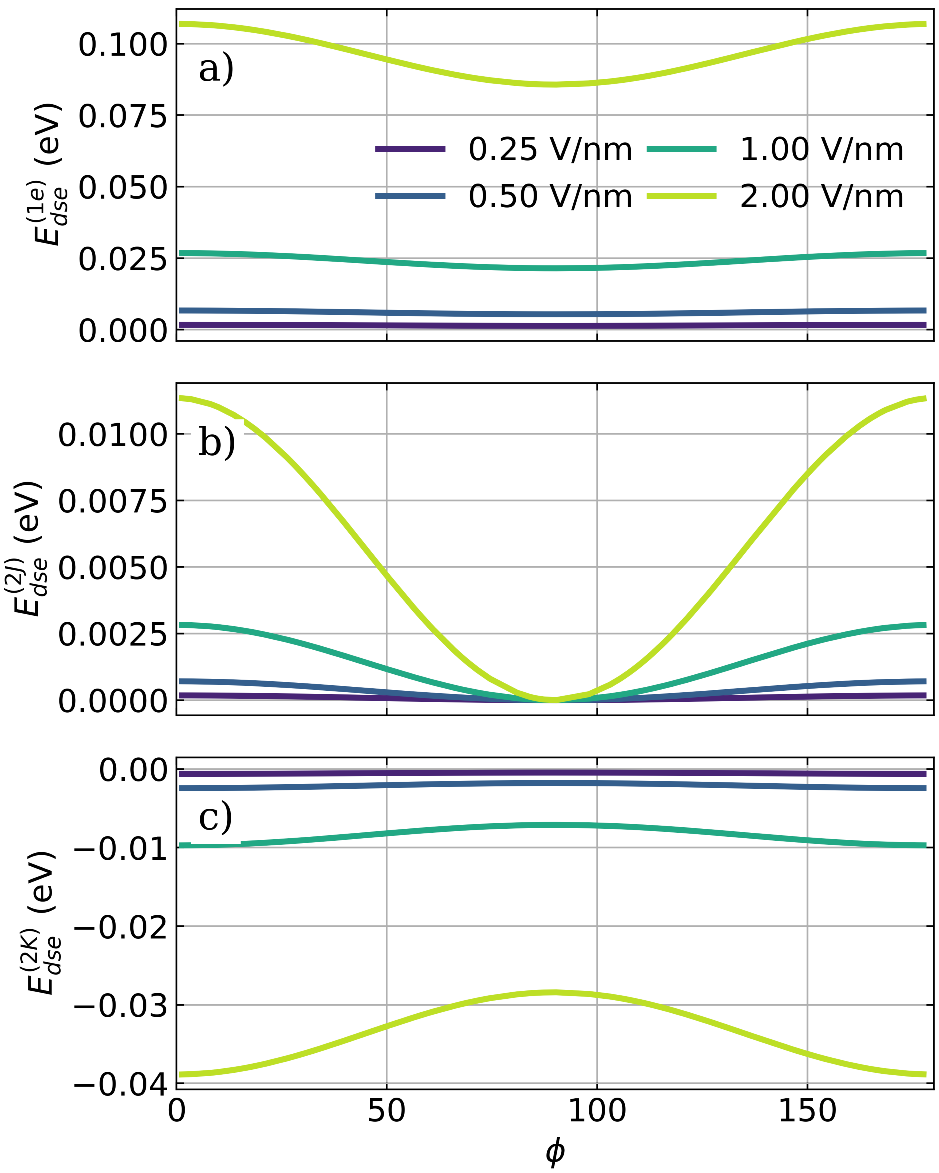

Figure S3: Scan along the angle ϕ, defined between $\boldsymbol{\mu}$ of a single HF and the polarization axis $\boldsymbol{e}$. a) $E_{dse}^{(1e)}$ b) $E_{dse}^{(2J)}$ c) $E_{dse}^{(2K)}$. for optimized q_{min}. All scans were performed with a cavity frequency ω_c of $4467\,\mathrm{cm^{-1}}$ and the cavity field strengths ϵ_c is increased from $0.25\,\mathrm{V\,nm^{-1}}$ to $2.0\,\mathrm{V\,nm^{-1}}$ (color-coded).

S3 Fixed ensembles of HF in an optical cavity

The change in individual molecular energy induced by the interaction with the cavity, as well as the underlying energy components, are visualized in Fig. S4 as a function of the size of the *all-parallel* ensemble N_{mol} without E_{dis} included.

Supplementary simulation results for fixed ensembles of HF molecules in an optical cavity without rescaling $\boldsymbol{\lambda}_c$ are shown in Figs. S5 and S6. The results discussed for the scaled case in the manuscript are still valid, and only the scaling behaviors change. For the ensemble perspective, see Fig. S5, the cavity-induced change in the total energy scales linearly with N_{mol}, while its three contributions (E_{dis}, E_{lin}, and E_{dse}) scale quadratic. The energy changes for an individual HF molecule, shown in Fig. S6 a), scales quadratic with N_{mol}. The linear interaction E_{lin} (Fig. S6 b)) and the interacting part of the E_{dse} (Fig. S6 d)) changing linear with N_{mol} and the local part of E_{dse} (Fig. S6 c)) is constant as expected.

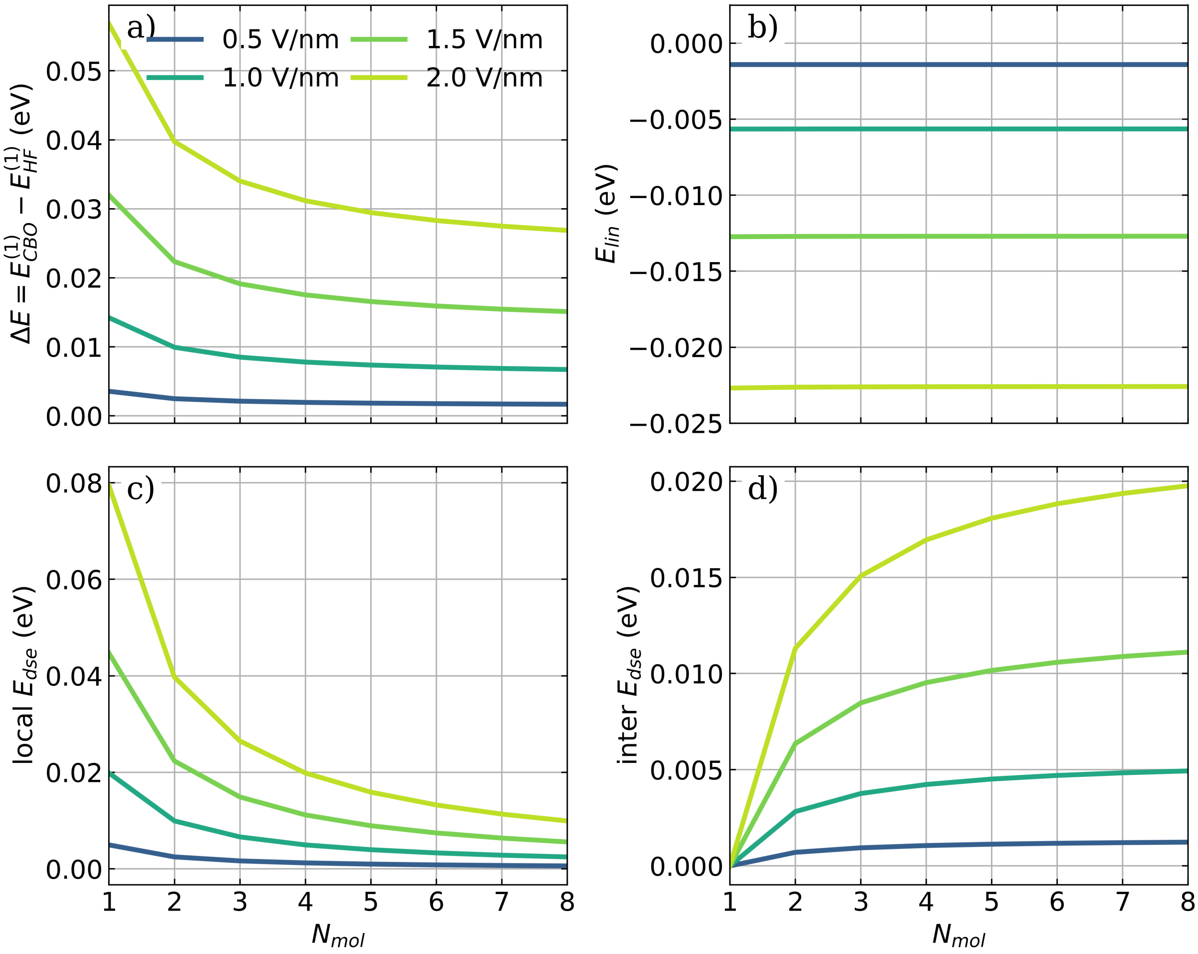

Figure S4: Influence of the cavity interaction on an individual HF molecule in *all-parallel* ensembles of different size and vacuum-field strengths ϵ_c. a) The energy difference ΔE between $E^{(1)}_{CBO}$ and the field-free energy $E^{(1)}_{HF}$ without E_{dis} included, b) the local linear energy contribution E_{lin}, c) the local E_{dse} and d) the intermolecular dipole-dipole energy as a function of N_{mol}. The individual dipole moments are aligned with the cavity polarization axis, and a cavity frequency ω_c of $4467\,\mathrm{cm}^{-1}$ is used. The strength of the cavity field ϵ_c increases from $0.5\,\mathrm{V\,nm}^{-1}$ to $2.0\,\mathrm{V\,nm}^{-1}$ (color-coded). The used coupling strength $\boldsymbol{\lambda}_c$ is rescaled according to Eq. 16.

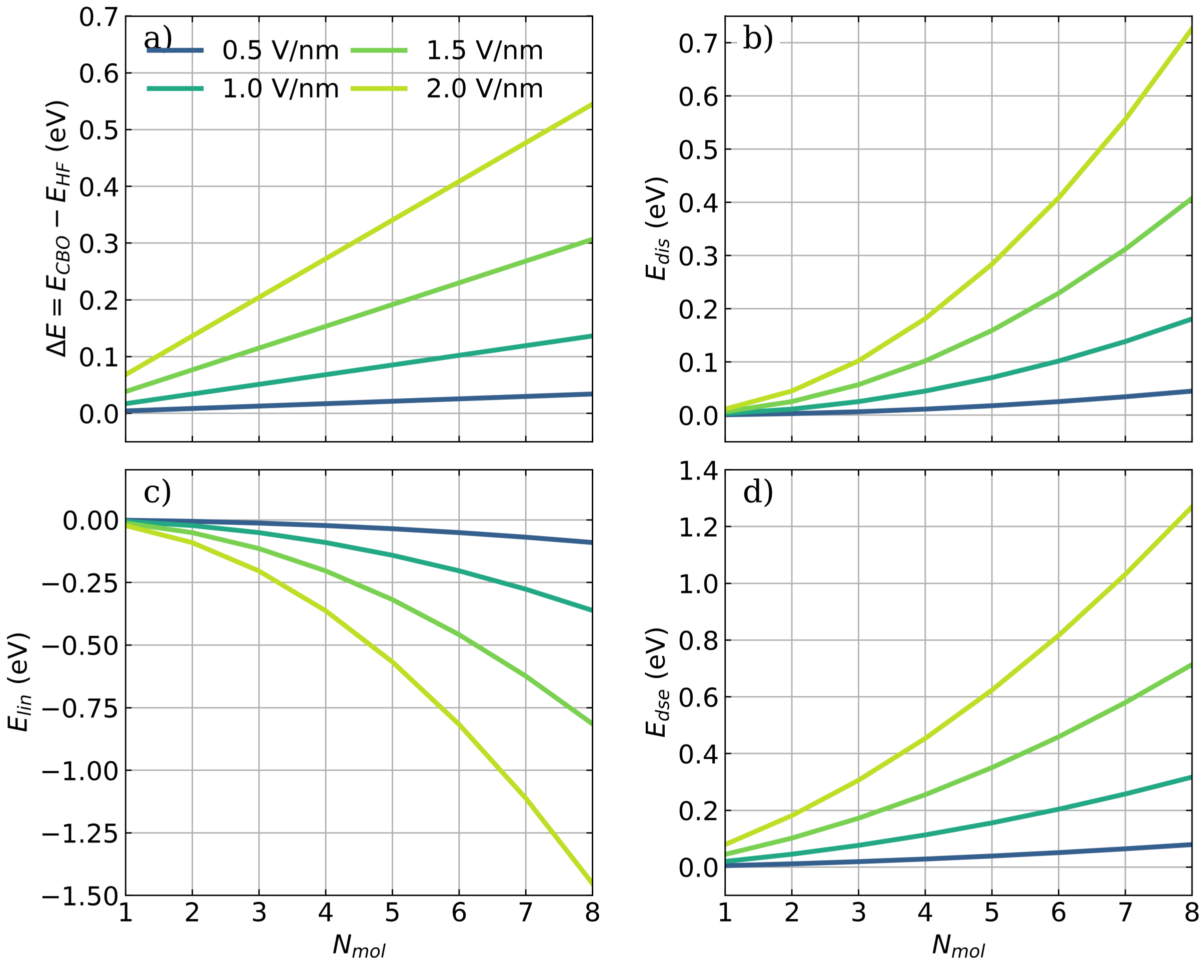

Figure S5: Influence of the cavity interaction on the collective energy of different ensembles of perfectly aligned HF molecules, without rescaling of $\boldsymbol{\lambda}_c$. a) The total energy E_{CBO} referenced to the case without a cavity (E_{HF}), b) cavity potential E_{dis}, c) linear energy contribution E_{lin}, and d) the DSE part E_{dse} for optimized q_{min} as a function of N_{mol}. Individual dipole moments are aligned with the cavity polarization axis and a cavity frequency ω_c of $4467\,\mathrm{cm}^{-1}$ is used. The strength of the cavity field ϵ_c increases from $0.5\,\mathrm{V\,nm}^{-1}$ to $2.0\,\mathrm{V\,nm}^{-1}$ (color-coded).

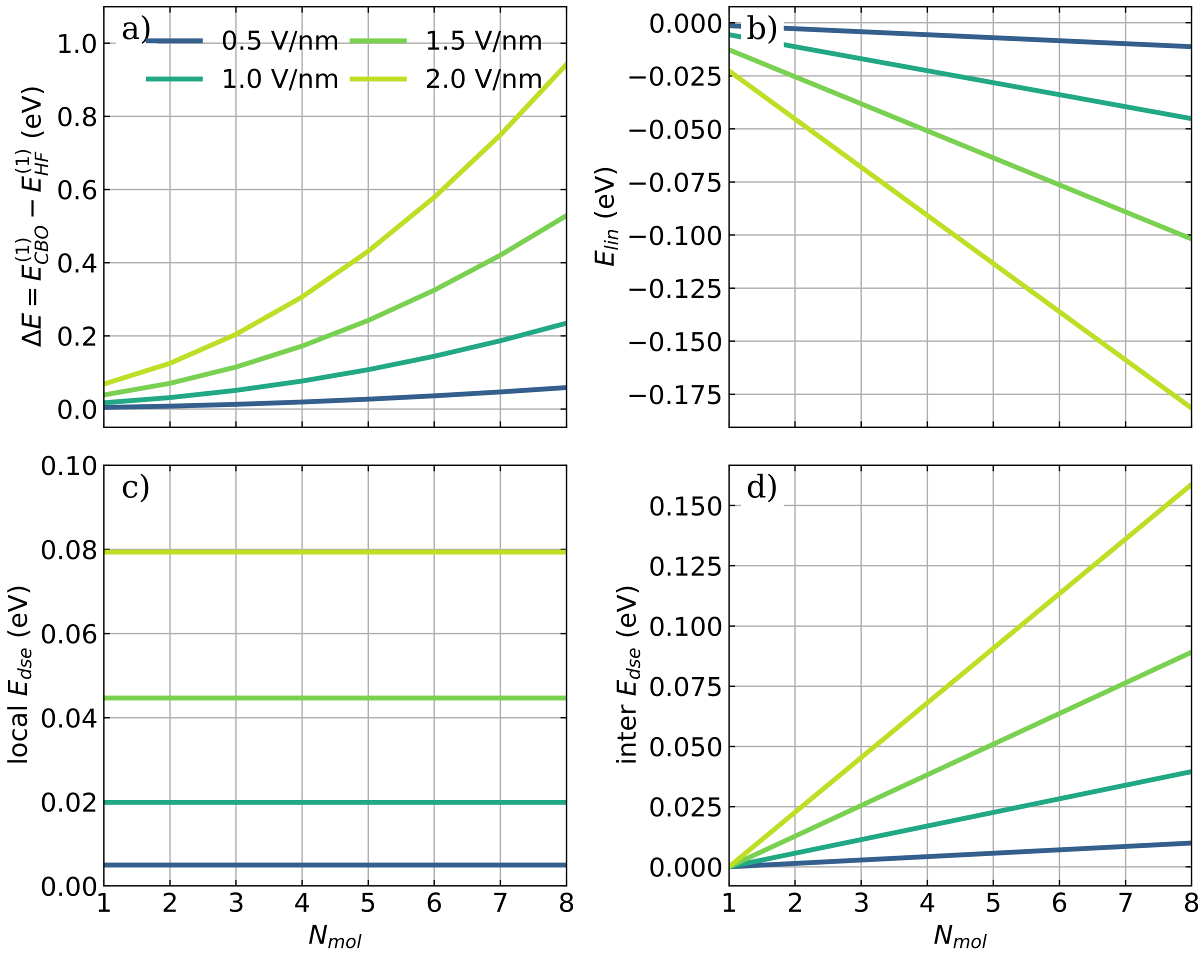

Figure S6: Influence of the cavity interaction on an individual HF molecule in ensembles of different size, without rescaling of $\boldsymbol{\lambda}_c$. a) The individual molecular energy E_{CBO} referenced to the case of a single molecule without cavity interaction, b) the local linear energy contribution E_{lin}, c) the local E_{dse} and d) the intermolecular dipole-dipole energy as a function of N_{mol}. The individual dipole moments are aligned with the cavity polarization axis and a cavity frequency ω_c of $4467\,\mathrm{cm}^{-1}$ is used. The strength of the cavity field ϵ_c increases from $0.5\,\mathrm{V\,nm}^{-1}$ to $2.0\,\mathrm{V\,nm}^{-1}$ (color-coded).

S4 Scanned ensembles of HF molecules in an optical cavity

Supplementary results for ensemble energy changes along the scan of a single HF bond are shown in Figs. S7, S8, and S9. Individual energy contributions for the dissociating HF molecule in different ensembles are shown in Figs. S10, S11, S12, and S13.

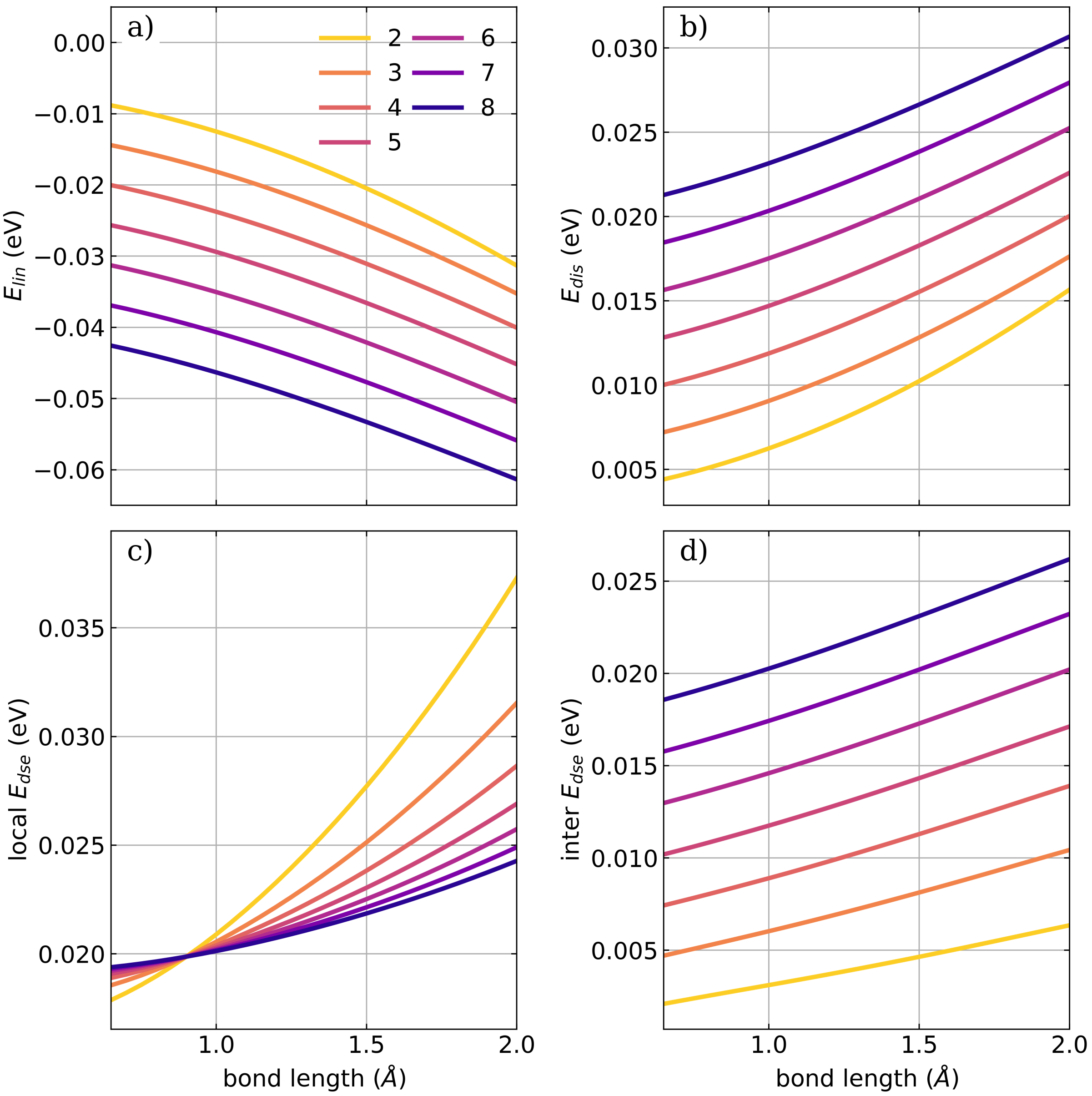

Figure S7: Cavity-induced energy contribution of the complete ensemble along the HF bond length for different ensemble sizes in the *all-parallel* configuration. a) linear energy contribution E_{lin}, b) cavity potential E_{dis}, c) local part of E_{dse} d) interaction part of E_{dse}. A cavity frequency ω_c of $4467\,\mathrm{cm}^{-1}$ is used. The strength of the cavity field ϵ_c is $1.5\,\mathrm{V\,nm}^{-1}$ and the number of molecules in the ensemble is color-coded.The used coupling strength $\boldsymbol{\lambda}_c$ is rescaled according to Eq. 16.

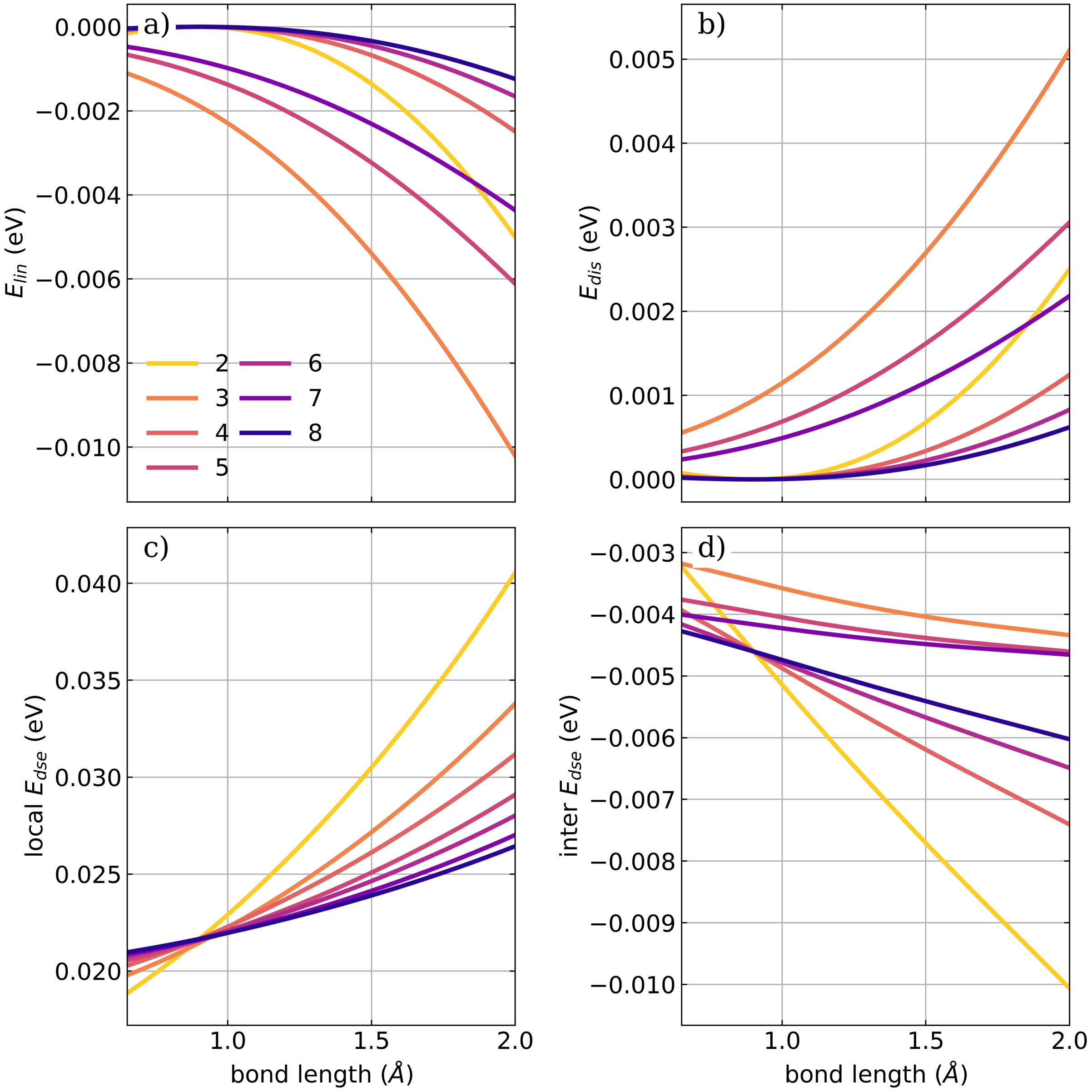

Figure S8: Cavity-induced energy contribution of the complete ensemble along the HF bond length for different ensemble sizes in the *antiparallel* configuration. a) linear energy contribution E_{lin}, b) cavity potential E_{dis}, c) local part of E_{dse} d) interaction part of E_{dse}. A cavity frequency ω_c of $4467\,\mathrm{cm}^{-1}$ is used. The strength of the cavity field ϵ_c is $1.5\,\mathrm{V\,nm}^{-1}$ and the number of molecules in the ensemble is color-coded.The used coupling strength $\boldsymbol{\lambda}_c$ is rescaled according to Eq. 16.

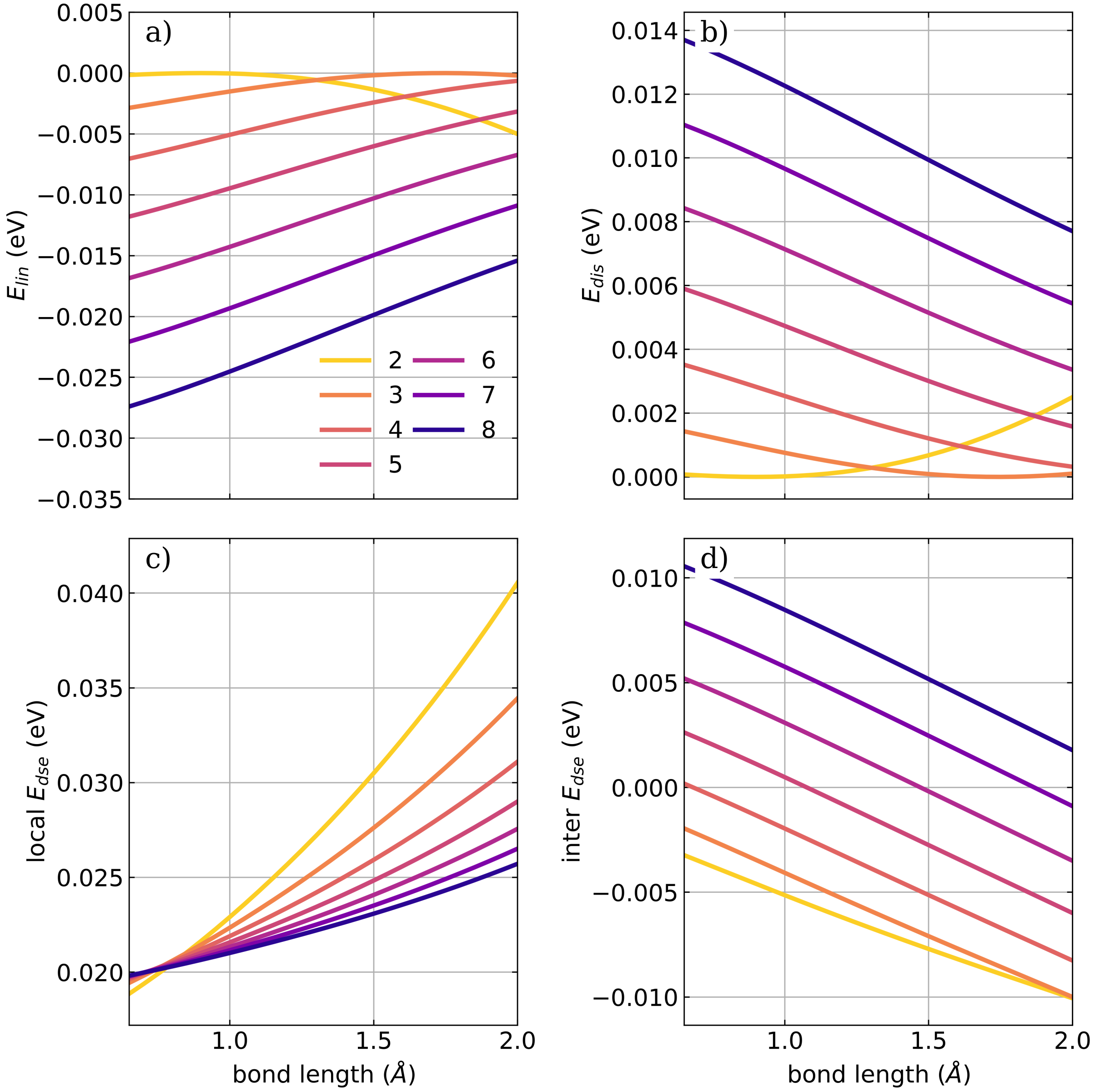

Figure S9: Cavity-induced energy contribution of the complete ensemble along the HF bond length for different ensemble sizes in the *defective* configuration. a) linear energy contribution E_{lin}, b) cavity potential E_{dis}, c) local part of E_{dse} d) interaction part of E_{dse}. A cavity frequency ω_c of $4467\,\mathrm{cm}^{-1}$ is used. The strength of the cavity field ϵ_c is $1.5\,\mathrm{V\,nm}^{-1}$ and the number of molecules in the ensemble is color-coded.The used coupling strength $\boldsymbol{\lambda}_c$ is rescaled according to Eq. 16.

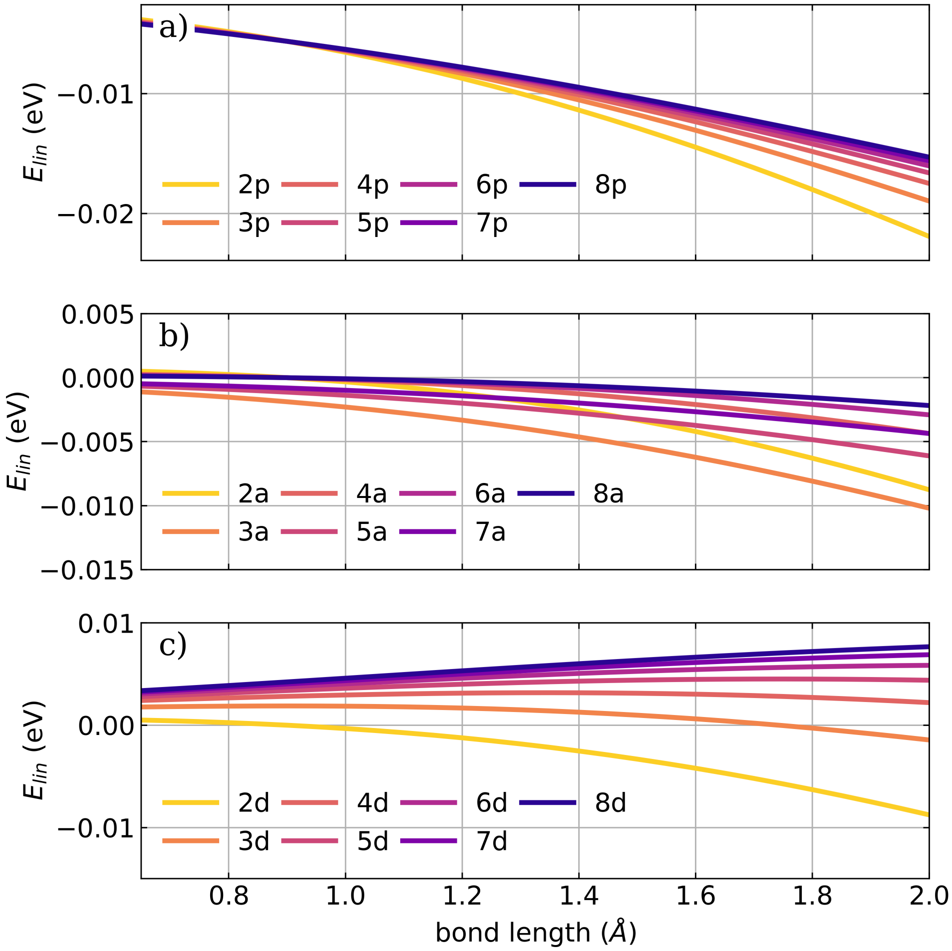

Figure S10: The local linear energy contribution E_{lin} along the HF bond length for different ensemble sizes is shown for a) the *all-parallel* configuration, b) the *antiparallel* configuration and c) the *defective* configuration. A cavity frequency ω_c of $4467\,\mathrm{cm}^{-1}$ is used. The strength of the cavity field ϵ_c is $1.5\,\mathrm{V\,nm}^{-1}$ and the number of molecules in the ensemble is color-coded.The used coupling strength $\boldsymbol{\lambda}_c$ is rescaled according to Eq. 16.

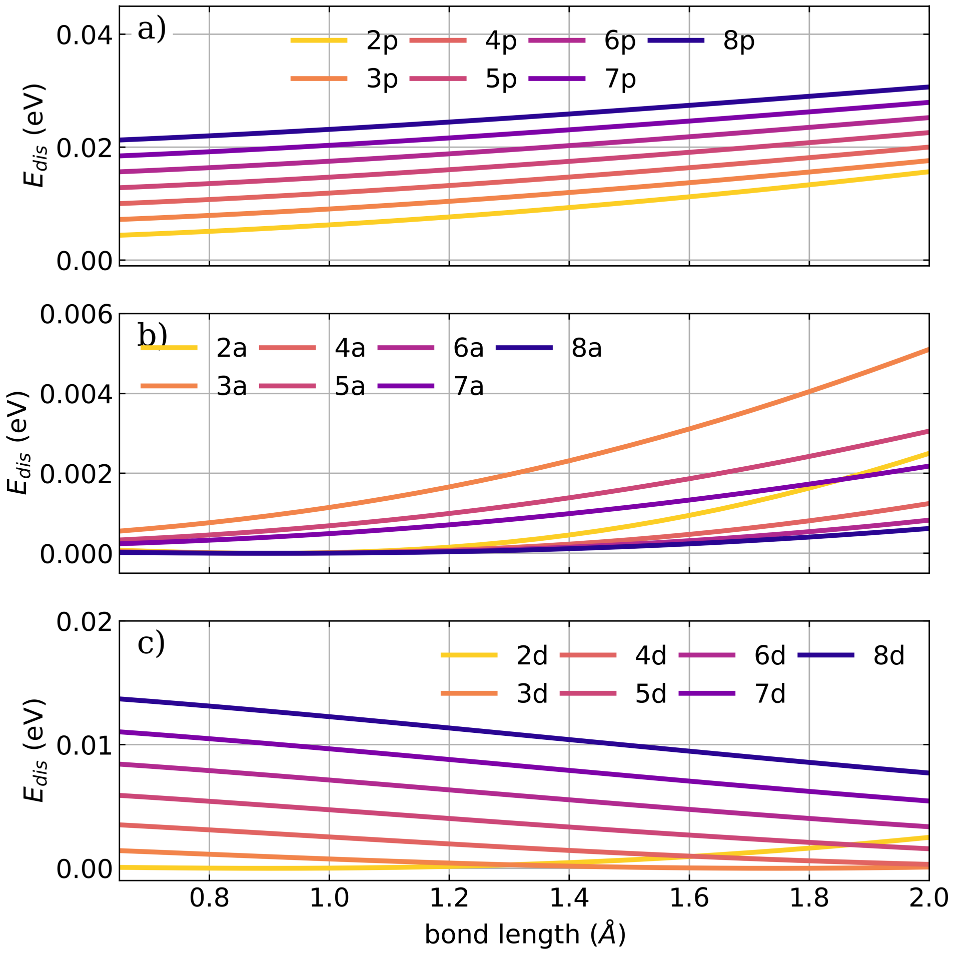

Figure S11: The cavity potential E_{dis} along the HF bond length for different ensemble sizes is shown for a) the *all-parallel* configuration, b) the *antiparallel* configuration and c) the *defective* configuration. A cavity frequency ω_c of $4467\,\mathrm{cm}^{-1}$ is used. The strength of the cavity field ϵ_c is $1.5\,\mathrm{V\,nm}^{-1}$ and the number of molecules in the ensemble is color-coded.The used coupling strength $\boldsymbol{\lambda}_c$ is rescaled according to Eq. 16.

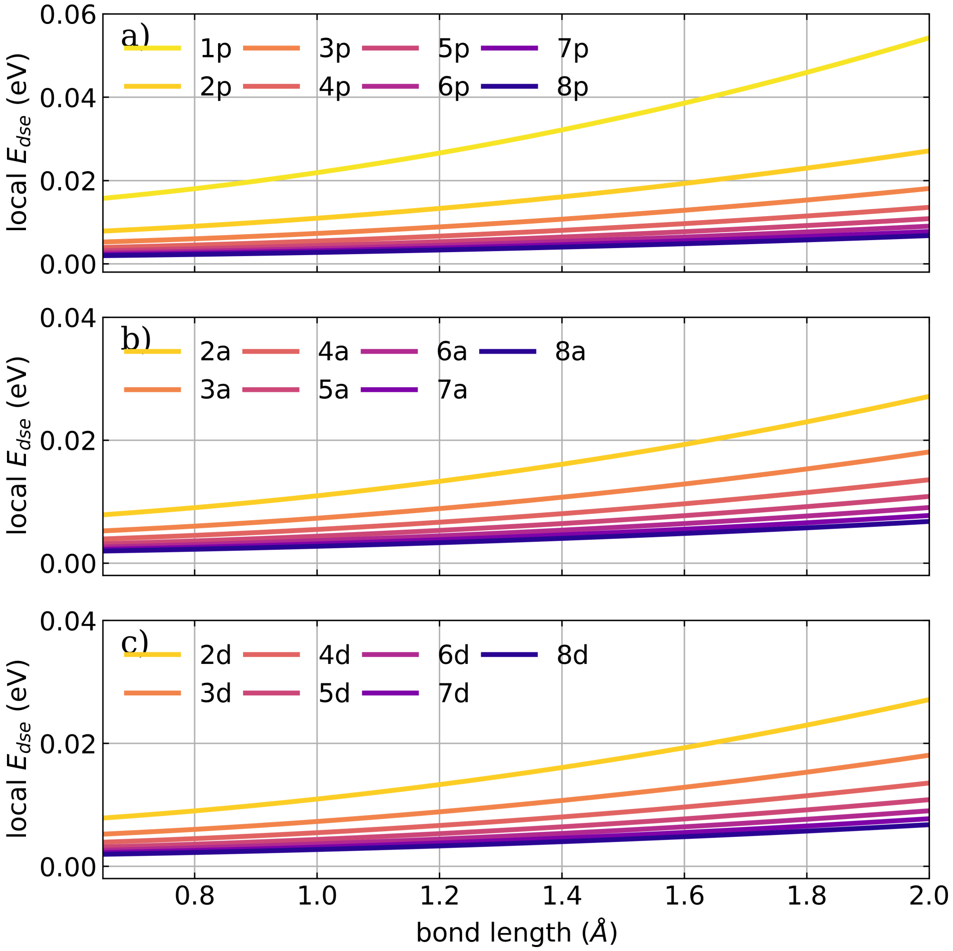

Figure S12: The local part of E_{dse} along the HF bond length for different ensemble sizes is shown for a) the *all-parallel* configuration, b) the *antiparallel* configuration and c) the *defective* configuration. A cavity frequency ω_c of $4467\,\mathrm{cm}^{-1}$ is used. The strength of the cavity field ϵ_c is $1.5\,\mathrm{V\,nm}^{-1}$ and the number of molecules in the ensemble is color-coded.The used coupling strength $\boldsymbol{\lambda}_c$ is rescaled according to Eq. 16.

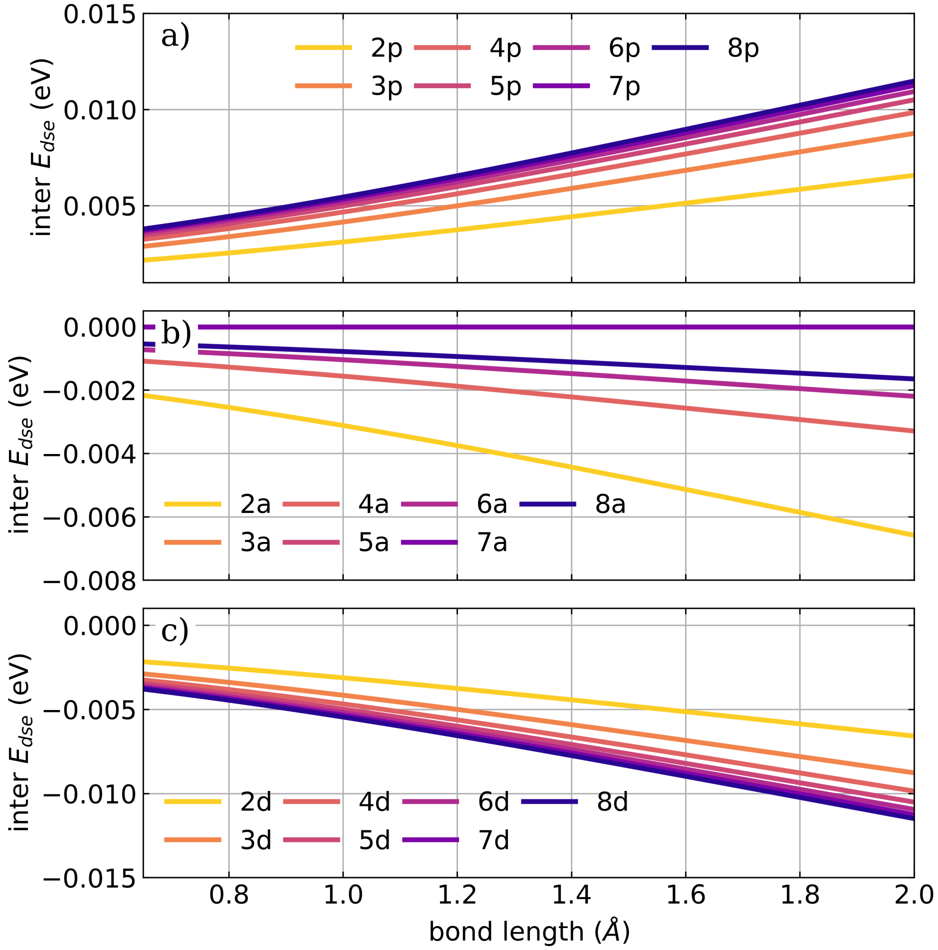

Figure S13: The local interaction part of E_{dse} along the HF bond length for different ensemble sizes is shown for a) the *all-parallel* configuration, b) the *antiparallel* configuration and c) the *defective* configuration. A cavity frequency ω_c of $4467\,\mathrm{cm}^{-1}$ is used. The strength of the cavity field ϵ_c is $1.5\,\mathrm{V\,nm}^{-1}$ and the number of molecules in the ensemble is color-coded.The used coupling strength $\boldsymbol{\lambda}_c$ is rescaled according to Eq. 16.

References

(1) Spohn, H. *Dynamics of charged particles and their radiation field*; Cambridge university press, 2004.

(2) Ruggenthaler, M.; Tancogne-Dejean, N.; Flick, J.; Appel, H.; Rubio, A. From a quantum-electrodynamical light–matter description to novel spectroscopies. *Nature Reviews Chemistry* **2018**, *2*, 1–16.

(3) Jestädt, R.; Ruggenthaler, M.; Oliveira, M. J.; Rubio, A.; Appel, H. Light-matter interactions within the Ehrenfest–Maxwell–Pauli–Kohn–Sham framework: fundamentals, implementation, and nano-optical applications. *Adv. Phys.* **2019**, *68*, 225–333.

(4) Lindoy, L. P.; Mandal, A.; Reichman, D. R. Quantum dynamical effects of vibrational strong coupling in chemical reactivity. *Nature Communications* **2023**, *14*, 2733.

(5) Flick, J.; Ruggenthaler, M.; Appel, H.; Rubio, A. Atoms and molecules in cavities, from weak to strong coupling in quantum-electrodynamics (QED) chemistry. *Proc. Natl. Acad. Sci. U.S.A.* **2017**, *114*, 3026–3034.

(6) Flick, J.; Appel, H.; Ruggenthaler, M.; Rubio, A. Cavity Born–Oppenheimer approximation for correlated electron–nuclear-photon systems. *J. Chem. Theory Comput.* **2017**, *13*, 1616–1625.

(7) Flick, J.; Narang, P. Cavity-Correlated Electron-Nuclear Dynamics from First Principles. *Phys. Rev. Lett.* **2018**, *121*, 113002.

(8) Szabo, A.; Ostlund, N. S. *Modern Quantum Chemistry: Introduction to Advanced Electronic Structure Theory*, 1st ed.; Dover Publications, Inc.: Mineola, 1996.
